# Cancer Extracellular Vesicles: Next-Generation Diagnostic and Drug Delivery Nanotools

**DOI:** 10.3390/cancers12113165

**Published:** 2020-10-28

**Authors:** Stefano Palazzolo, Lorenzo Memeo, Mohamad Hadla, Fahriye Duzagac, Agostino Steffan, Tiziana Perin, Vincenzo Canzonieri, Tiziano Tuccinardi, Isabella Caligiuri, Flavio Rizzolio

**Affiliations:** 1 Pathology Unit, Centro di Riferimento Oncologico di Aviano (CRO) IRCCS, 33081 Aviano, Italy; spalazzolo@cro.it (S.P.); tperin@cro.it (T.P.); vcanzonieri@cro.it (V.C.); 2 Department of Experimental Oncology, Mediterranean Institute of Oncology, 95029 Catania, Italy; lorenzo.memeo@grupposamed.com; 3 Biofield Innovation, 35127 Padova, Italy; hadla@abanalitica.it; 4 Department of Molecular Sciences and Nanosystems, Ca’ Foscari University, 30170 Venezia-Mestre, Italy; fahriye.duzagac@unive.it; 5 Immunopathology and Cancer Biomarkers, Centro di Riferimento Oncologico di Aviano (CRO) IRCCS, 33081 Aviano, Italy; asteffan@cro.it; 6 Department of Medical, Surgical and Health Sciences, University of Trieste, 34149 Trieste, Italy; 7 Department of Pharmacy, University of Pisa, 56126 Pisa, Italy; tiziano.tuccinardi@unipi.it

**Keywords:** extracellular vesicles, microvesicles, diagnostics, drug delivery, nanomedicine, cancer

## Abstract

**Simple Summary:**

Extracellular vesicles (EVs) are secreted continuously from different cell types. The composition of EVs, like proteins, nucleic acids and lipids is linked with the cells of origin and they are involved in cell-cell communication. The presence of EVs in the majority of the body fluids makes them attractive to investigate and define their role in physiological and in pathological processes. This review is focused on EVs with dimensions between 30 and 150 nm like exosomes (EEVs). We described the biogenesis of EEVs, methods for isolation and their role in cancer as innovative diagnostic tools and new drug delivery systems.

**Abstract:**

Nanosized extracellular vesicles (EVs) with dimensions ranging from 100 to 1000 nm are continuously secreted from different cells in their extracellular environment. They are able to encapsulate and transfer various biomolecules, such as nucleic acids, proteins, and lipids, that play an essential role in cell‒cell communication, reflecting a novel method of extracellular cross-talk. Since EVs are present in large amounts in most bodily fluids, challengeable hypotheses are analyzed to unlock their potential roles. Here, we review EVs by discussing their specific characteristics (structure, formation, composition, and isolation methods), focusing on their key role in cell biology. Furthermore, this review will summarize the biomedical applications of EVs, in particular those between 30 and 150 nm (like exosomes), as next-generation diagnostic tools in liquid biopsy for cancer and as novel drug delivery vehicles.

## 1. Introduction

Cancer is a severe worldwide health problem. In order to improve the survival rate of patients, clinicians and medical researchers have worked on early diagnosis and awareness of risk factors for cancer. In the meantime, we cannot deny the urgent need for novel markers with better efficiency and less invasive features, as these markers could lead to early diagnosis, changing the therapeutic direction for individual patients with a more precise estimation of prognosis. Cell-derived materials are considered as specific markers that reflect definite cellular characteristics, and extracellular vesicles (EVs) in particular are attracting a lot of interest when it comes to cancer diagnosis and treatment since different cell types produce distinctive amounts of EVs. EVs can be divided into a few subgroups based on their biogenesis, size, and biomarkers. In particular, three main group have been described: intraluminal vesicle (ILV)-derived EVs; microvesicles, and apoptotic bodies. It should be highlighted that most studies have not clearly described the origin of the EVs under investigation. It should also be underlined that the subcellular origin of EVs is often not described in most of the published literature; EVs are differentiated based on the size and expression of biomarkers that we now know are often not exclusive to a particular EV class. Due to this, in many cases, it is not possible to assume that these studies were indeed looking at ILV-derived EVs rather than other biogenesis pathways, but were small EVs of an undetermined origin. In this review, we will focus our attention on those EVs with a size between 30 and 150 nm, like exosomes; we will call these exosome extracellular vesicles (EEVs) throughout the text.

The first description of EEVs’ secretion was mentioned in reticulocytes, platelets, dendritic, lymphocytes B and T, mast cells, and macrophage hematopoietic cells [1,2,3,4,5,6,7,8,9,10,11]. Additionally, other nonhematopoietic cells can produce EEVs, such as astrocytes, melanocytes, neurons, adipocytes, epithelial, fibroblasts, and tumor cells [12,13,14,15,16,17,18,19,20]. EEVs are present in most of the physiological fluids, including blood, serum, urine, saliva, breast milk, lymph, amniotic fluid, ascites, semen, cerebrospinal fluid, and nasal secretions [21]. EEVs play a key role in short- and long-distance cell communication, promoting the development and function of multicellular organisms under physiological and pathological conditions. EEVs are deeply involved in long-distance communications to transfer proteins [22,23,24,25], mRNAs, and miRNAs that could be expressed in target cells [26,27]. This mechanism ensures highly efficient secretion, signaling, and communication, in a robust and economic manner, for information exchange between cells [28]. Depending on the cellular origin, EEVs can contain different profiles of RNA, miRNA, and proteins, including tetraspanins, metalloproteinases, major histocompatibility molecules (MHC), and adhesion molecules [20,29,30]. These molecules can be altered by stress or pathological conditions; thus, molecular profiles of circulating EEVs can be used for theragnostic implications. Cancer-derived EEVs are ideal as biomarkers for the early diagnosis of cancer as they carry specific molecules that reflect the genetics and signaling alterations of parental cancer cells [31,32,33]. 

As well as being a potential diagnostic tool, EEVs are ideal drug delivery vehicles. A size of about 100 nm has been demonstrated to be optimal for a long circulating time in biological fluids, avoiding fast elimination [34]. The EEV membrane contains a specific set of lipids and proteins similar to that of the cell of origin, with infinite combinations of targeting possibilities. The membrane structure and composition allow for immune surveillance escape, ideal interaction with cell membranes, and internalization, better than formulated pegylated liposomes [35,36,37,38]. EEVs are mediators of cell communication [27,39,40] and many research groups, including us, have utilized EEVs to deliver nucleic acids, proteins, or small molecules [41,42,43,44]. 

In the first part of this review, after a brief introduction to EEVs’ biology, we point out the attention to cancer-derived EEVs as potential biomarkers, which will probably have a high impact in the near future in terms of facilitating the early detection, monitoring, and prognosis of cancer. In the second part, we focus on the use of EEVs as novel drug delivery systems, depending on their specific features to apply them as anticancer therapeutic vehicles in vitro and in vivo, and discuss the limits and the prospective challenges.

### 1.1. EEVs’ Biogenesis

**Figure 1:**
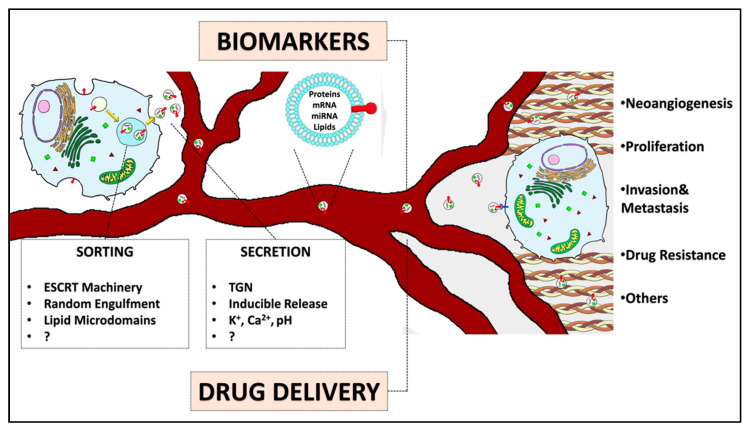
EEVs’ biogenesis, content, and functions. EEVs are eventually secreted by any cells in the bloodstream to deliver their cargos to targeted cells, regulating, among other functions, neoangiogenesis, proliferation, invasion, metastasis, and drug resistance. EEVs are demonstrated to be useful biomarkers for cancer diagnosis and prognosis. These nanovesicles could also be engineered in order to be loaded with drugs or therapeutic molecules.

During their life cycle, eukaryotic cells periodically engulf small amounts of intracellular fluids and form small intracellular bodies called endosomes [29,45]. As the early endosome is maturing into a late endosome, it forms ILVs in the lumen of endosomes. ILVs have a range of 30–100 nm of diameter and are formed by inward budding of the endosome’s membrane. Portions of the cytosol and incorporated transmembrane/peripheral proteins are engulfed into the invaginating membrane. This phenomenon can be detected in late endosomes by following the changes in their location and shapes: early endosomes are located in the outer part of the cytoplasm with a tube-like shape, whereas late endosomes are present near to the nucleus with a spherical shape [29]. Late endosomes containing ILVs are also called multivesicular bodies (MVB) [46,47]; usually, they fuse with lysosomes, followed by degradation of their contents mainly by hydrolysis. It has been hypothesized that MVBs’ content can be divided based on their function: the proteins found in the ILVs are destined for lysosomal degradation, while functional proteins with a biological role are imported into the ILVs of MVB [48]. Until now, the mechanism that describes this process has not been completely understood, and there are different theories about protein sorting [49]. MVBs may fuse with the plasma membrane instead of fusion with lysosomes and therefore release ILVs to the extracellular space (Figure 1); these released vesicles are then called EEVs [46,50].

The uniformity of EEVs originating from similar cell types reveals the presence of a sorting mechanism behind EEVs’ development. The protein sorting of ILVs in MVBs is thought to involve different participants such as an endosomal sorting complex required for transport (ESCRT) components (e.g., Alix, Tsg101, and clathrin), lipids, and/or tetraspanin-enriched microdomains present in EEVs. The sorting mechanism started with the recognition of the ubiquitinated cargo proteins by an ESCRT complex, which then recruits the ESCRT-I subunit Tsg-101, activating AIP/Alix [51]. All these sequential interactions drive the cargo into the budding vesicles. It appears that there is also an ESCRT-independent mechanism involved in protein sorting to MVBs [52]. The sorting of cytosolic proteins does not require the ESCRT machinery and can be explained by a “random” engulfment of small portions of cytosol during the inward budding process and/or by their transient association with transmembrane proteins [53]. Moreover, the sequestration of glycophosphatidylinositol-anchored proteins and other “raft”-associated proteins in EEVs reflects the presence of lipid-like domains in EEVs’ membranes. The lipid microdomains could themselves be involved in the generation of ILVs, or in concert with other proteins, with affinity for “raft-like domains” such as tetraspanins [46,54]. The biogenesis of EEVs indicates that their macromolecule composition, including proteins, lipids, mRNAs, and microRNAs, can be varied depending on the cell of origin. Protein analysis of EEVs from different cell types including dendritic cells (DC) [47], B-lymphocytes [7], and epithelial intestinal cells [55] revealed that there are common, as well as cell-type-specific, proteins within EEVs. Common proteins shared by various EEVs are Annexins I, II, V, and VI, which could be involved in cytoskeleton dynamics and membrane fusion [10,56]. The Ras superfamily of monomeric G proteins (Rab) also contains common proteins that could act in EEVs’ docking and on the ability to fuse with membranes of other cells [10,57]. Adhesion molecules [10], apoptosis proteins, heat shock proteins (Hsc73 and Hsc90), tetraspanins (CD9, CD63, CD81, and CD82 [47,55,58,59]), GTPases, and cytoskeletal proteins (actin, synenin, moesin, and albumin) [60] are classically found within EEVs of different origins. Other main EEV proteins are reported in Table 1.

It was demonstrated in vitro and in vivo that EEVs are produced in both normal and pathological conditions [64]. EEVs have been involved in numerous physiological processes, including the removal of unnecessary proteins from cells, but their principal role is in cell‒cell communication, both locally and systemically, by transferring their contents, including protein, lipids, and RNAs, between cells [65]. The role of EEVs in immune system stimulation was extensively investigated [66]; it was also proposed a new approach to vaccine development [67]. EEVs were described as playing a pivotal role during normal development and the physiology of the nervous system, acting as cell–cell communicators and playing functional roles not only during development but also during the regeneration of normal neurons [68]. Besides their physiological role, EEVs were depicted as a Trojan horse in neurodegenerative processes due to their capability to transfer “toxic” cargos from unhealthy to normal neurons [69,70]. Recently, it was demonstrated that EEVs are involved in a wide range of cardiovascular physiological and pathological processes, with beneficial or pathological activity [71,72]. In this review, we discuss the EEVs’ role in cancer. Cancer cells have shown a higher secretion rate of EEVs compared to normal cells [73,74]. The secretion of EEVs in cells could follow two different mechanisms: the constitutive secretion involving the trans-Golgi network (TGN) and/or inducible release, depending on the cell type and on the activation state of the cell [75]. The constitutive pathway does not require a specific stimulus, although it is controlled by cell activity (intracellular signaling, cell growth, differentiation, DNA damage, etc.). Proteins destinated to be secreted into the extracellular medium or to the cell surface can be routed from the TGN, where EEVs are transported within vesicles containing only one or two EEVs. However, the inducible release of EEVs requires specific stimuli such as hypoxia or toxic stress, causing DNA damage and leading to vesicular trafficking [29]. Thery et al. have demonstrated that Rab proteins such as Rab27 isoforms play a regulatory role in EEVs’ secretion [76]; in addition, Rab35 family members have been shown to be an essential part in the regulation of EEVs’ secretion due to the interaction with the TBC1 domain of GTPase-activating protein and 10A-C (TBC1D10A-C) family members [77,78]. It is known that Rab proteins are usually mutated (constitutively active) or overexpressed in tumor cells [79]. Furthermore, other studies have shown that, due to the activation of the tumor suppressor protein p53, EEVs’ secretion rate is stimulated by regulating the transcription of different genes like TSAP6 and CHMP4C [75,80,81]. Once the cells suffer from stresses like toxicity or hypoxia, damage may occur at the DNA level; then, a response of the p53 protein is generated by regulating the transcription of different genes [82]. This process is also called the “bystander effect”, in which cells communicate with the microenvironment by the secretion of specific proteins in order to compensate for a response to stress [83]. Evidence of this process was seen during the irradiation of human prostate cancer cells, which led to DNA damage that could induce an increase in EEV production due to cell activation [84]. Many other mechanisms are correlated to EEVs’ secretion in several cell types, including K^+^ depolarization of neuronal cells, intracellular Ca^2+^ level, pH variation, and CD3 crosslinking with T cells [29,46,83,85]. 

Various functions are carried out by EEVs once they are secreted from the cells of origin and might be transferred to other cells [86]. It has been shown that some EEVs, such as those secreted by tumor cells, were found to carry phosphatidylserine (PS) on their surface as signal transduction, allowing their uptake by appropriate cells via specific mechanisms [87,88]. Once EEVs are taken up by other cells, they can be either endocytosed via clathrin-coated pits or could release their contents in the cell and remain joined with the plasma membrane [89]. EEVs contain various molecules derived from the cell surface, which allows them to recognize different cell receptors at the same time [90]; furthermore, the EEV‒cell interaction favors the intercellular exchange of several materials like lipids, proteins, carbohydrates, and pathogens. Recent data from 286 studies on the ExoCarta database show that 9769 proteins, 3408 mRNA, 2838 miRNA, and 1116 lipids (07/10/2015 Exocarta update published in *J Molecular Biology* [91]; http://www.exocarta.org/) are associated with EEVs, thus demonstrating the complexity of EEVs.

### 1.2. EEVs’ Isolation Methods

Size, protein and lipid contents are usually used to characterize EEVs and related microvesicles [28]. The complete separation and purification of each type of vesicles would help to exploit their benefits for clinical use; however, this is extremely hard if not impossible [37,92]. Researchers are trying to overcome these limits, although the absolute separation and definition of various extracellular vesicles and EEVs based on their size or biogenesis is still to be determined because no markers could distinguish the origin of the EEVs [93], and there are difficulties in sorting them due to their heterogeneous biochemical composition [94]. EEVs could be isolated from body fluids or from processed cell culture media through different techniques (Table 2), based on EEV surface markers such as tetraspanins, integrins, and cell adhesion molecules [95], or lipid composition, as they are rich in cholesterol, phosphatidylcholine, and phosphatidylethanolamine [96]. These techniques include immunoaffinity or size-exclusion chromatography (SEC), differential centrifugation, filtration coupled with centrifugation, microfluidic technologies, and polymer-based precipitation [28]. With biological fluids or cell culture supernatants that may be the source of EEV, the initial volumes can be scaled up or down according to the number of vesicles required for further analysis. Although biological fluids contain a large number of exosomes, they also have large numbers of soluble proteins and aggregates content, which could lead to contamination issues during EEV isolation methods.

Differential ultracentrifugation and density-gradient centrifugation are the most important methods for isolating EEVs [97]. In the differential ultracentrifugation method, different centrifugal forces are applied sequentially to a solution containing EEVs, eliminating cells, dead cells, cellular debris, intact organelles, and, finally, EEVs. One limit of this technique is that other vesicles and proteins can be deposited. Moreover, even in this sequentially extended ultracentrifugation, contaminants from bovine small RNAs could be miscategorized as human RNA. Density-gradient centrifugation can help to overcome this limitation: using a sucrose density gradient, contaminants may be separated from EEVs, resulting in a more uncontaminated fraction [98]. Another technique is immunoaffinity chromatography, which depends on antibody recognition of EEV proteins: antibodies are covalently attached to beads, binding specifically to surface proteins or antigens on the EEV surface, which helps to minimize antibody contamination and buffers interference [93].

Polyethylene glycol (PEG) solutions have been used to precipitate and isolate viruses and other macromolecules for more than five decades. EEVs are typically isolated using a precipitation solution consisting of PEG with a molecular weight of about 8 kDa. This solution is mixed with the source containing EEVs and then centrifuged at a low speed to form a pellet containing EEVs, which still carry a risk of contamination by proteins, before collecting the pellet from the upper aqueous phase [99].

Size-exclusion chromatography is a different method used to separate heterogeneous populations of different vesicle size in a solution. Biological materials with a small radius can penetrate through pores, whereas larger components, such as EEVs, are unable to pass through the pores [100]. Evidence showed that for the lipidomics profiling of plasma- or serum-derived EEVs, size-exclusion chromatography could be the most suitable method due to their porous structure reducing the risk of contamination with undesired circulating lipids and biological fluids [101].

Immunoaffinity approaches using antibodies specific for EEVs surface proteins could be integrated with microfluidic technologies. These antibodies are covalently bound to the chip for the separation of EEVs from other contaminants [102]. This allows for the rapid production and isolation of EEVs, but not in sufficient quantities to enable their use in the clinic. Chip-based immunoaffinity was developed to isolate EEVs and microvesicles, allowing for quantitative and high-throughput analysis of EEVs’ contents [103]. A microfluidic device formed by a porous silicon nanowire on the micropillar structure can trap EV-like lipid vesicles while filtering out proteins and debris. Extracellular vesicles are sieved through a porous membrane with a specific size, and collected by filtering the biofluid through a membrane; the filtration is then driven by either pressure or electrophoresis to assist EEVs’ separation from contaminants. Proteins are less affected by the electric field due to their lower negative charge compared to phospholipidic vesicles [104]. A porous ciliated silicon microstructure could selectively trap particles of 40–100 nm in size. This technique offers benefits in terms of both diagnostic and therapeutic applications [105]; however, stronger collaborations between microfluidic engineers and clinicians would be able to take advantage of microfluidic technology to isolate EEVs from body fluids [102]. It is well known that EEVs have significant advantages for disease diagnostics and monitoring because of their abundance, stability, and unique molecular cargos [106]. Since the challenges of EEVs’ isolation could be addressed by the integration of a microfluidics platform, clinicians must contribute to point-of-care testing and treatment options by focusing on these potential players.

## 2. EEVs in Tumor Malignancy

EEVs have many roles in natural and pathological processes. They were identified as mediators for reticulocyte maturation and, since then, investigators have shown the ability of EEVs to eliminate nonessential proteins and unnecessary molecules from the cell and suggested that EEVs are involved in cellular communications, intercellular material exchange, pathogen spreading, immune system regulation, and many other functions [107,108,109,110]. Thus, the functions of EEVs are varied and depend on their cell type of origin, which enables them to provide extracellular communications between normal and malignant physiological conditions (Table 3). These extracellular communications are also important factors for tumor malignancies, regulating cell proliferation, migration, invasion, survival, and metastasis. EEVs contribute to the complexity of the tumor microenvironment (TME). EEVs derived from tumor and stromal cells (macrophages, mesenchymal stem cells (MSCs), and fibroblasts) could affect each other and act as protumorigenic factors by triggering intracellular signal transduction mechanisms. In the TME, EEVs accelerate angiogenesis by secreting factors that act on the stromal cells to either promote or inhibit the growth of new blood vessels [111]. EEVs may have a high level of pro-angiogenesis molecules, promoting endothelial cell proliferation and the survival and differentiation of fibroblasts into myofibroblasts, leading to tumor vascularization. They can also enhance vessels’ permeability, leading to tumor cell diffusion and metastasis [112]. Interest has grown in adipose-derived stem cells in the TME; preclinical studies suggested that adipose-derived stem cells could be a potential tumor promoter, supporting tumor progression and activating several intracellular signals [113]. It is also important to consider tumor-associated macrophage (TAM)-derived EEVs’ role in promoting tumor progression and metastasis [114]. Tumor cell-associated EEVs can also induce the reprogramming of fat-derived stem cells to undergo neoplastic transformation and expansion of tumor cells. EEVs may alter the TME and prepare a substructure for distant tissue metastases and adhesion processes [115]. Finally, EEVs from tumor and stroma cells may reach the lymph nodes and distant organs, establishing premetastatic niches for metastatic colonization and suppression of the host immune response, and they may also deliver matrix metalloproteinases (MMPs) to degrade the extracellular matrix (ECM), affecting cell adhesion [116,117,118].

### 2.1. Cancer EEVs Promote Neoangiogenesis

Neoangiogenesis is an important step in cancer growth as it allows blood supply for tumor expansion and dissemination. EEVs derived from tumors may promote the differentiation of fibroblasts and adipose tissue-derived MSCs, influencing tumor angiogenesis [147]. Moreover, hypoxia caused by the reduction of tissue oxygen induces the secretion of angiogenic and metastatic factors through EEVs by tumor cells [124]. EEVs derived from glioblastoma multiforme (GBM) cells reveal hypoxic conditions and activate several cell surface receptors, which elicits an angiogenic response [148]. EEVs from K562 [120] and LAMA84 [107] chronic myelogenous leukemia (CML) cell lines, when internalized by human umbilical vein endothelial cells (HUVECs) during tubular differentiation, induced angiogenic effects. Other EEVs from human CD34-positive stem cells containing high levels of pro-angiogenic miRNAs (miR-126 and miR-130a) conferred in vivo angiogenesis ability and led to the in vitro formation of vessel-like endothelial structures of HUVECs [122]. EEVs derived from a gastric cancer patient contained miRNA155, which negatively regulates C-MYB and in turn promotes angiogenesis via VEGF [123]. The CD105-positive EEVs expressing a set of pro-angiogenic miRNAs, such as VEGF, FGF2, MMP-2, and MMP-9, mediate an angiogenic phenotype and induce vessel formation in SCID mice [121]. EEVs expressing carbonic anhydrase 9, a hypoxia inducible enzyme, were also found to promote angiogenesis [149].

### 2.2. Cancer EEVs Promote Invasion and Metastasis

Fibroblasts and inflammatory cells are tumor-associated stromal cells that can produce EEVs to deliver proteins or miRNAs into adjacent tumor cells. EEVs produced by fibroblasts stimulated invasive behaviour and metastasis of breast cancer cells [150]. It was also reported that EEVs from IL-4-activated macrophages induce invasiveness of SKBR3 and MDA-MB-231 breast cancer cells in vitro [130]. Additionally, EEVs isolated from the pluripotent bone marrow MSCs of patients with multiple myeloma (MM) have a lower amount of the tumor suppressor miR-15a and higher amounts of oncogenic proteins when compared to the EEVs derived from normal individuals. These EEVs promote tumor growth and induce dissemination of tumor cells to the bone marrow in an animal model of MM, whereas EEVs derived from normal bone marrow MSCs had the opposite effect on tumor cells [151]. Another study showed that EEVs derived from human bone marrow MSCs enhance VEGF expression in tumor cells by activating the ERK1/2 pathway. MSC-derived EEVs, subcutaneously injected in mice with gastric cancer cells, promote tumor growth and increase tumor blood supply [152]. Liang et al. showed that platelet-derived EEVs from patients with hematogenous metastatic lung cancer are enriched in miR-223, which promotes lung cancer cell invasion [153]. In 2018, Song et al. demonstrated that EEVs containing elevated CXCR4 (CXC chemokine receptor-4) derived from high lymph node metastatic mouse hepatocarcinoma Hca-F cells were able to promote the migration and invasion of paired syngeneic Hca-P cells that have low metastatic potential. It was demonstrated that the metastatic potential is increased by EEVs derived from Hca-F cells [127]. The importance of TAM-derived EEVs was described by Zheng et al. in 2018 [114]. They described the effect of TAM-derived EEVs on the migration of gastric cancer cells (GC). They found that gastric TAMs were predominantly constituted by a macrophage subpopulation promoting protumorigenic activity called M2. They found that M2 EEVs promoted the migration of GC in vitro and in vivo.

### 2.3. Cancer EEVs Promote Prometastatic Niche Formation and ECM Degradation

EEVs delivered into the extracellular environment can travel to surrounding cells and reach distant sites, such as the bone marrow, lungs, lymph nodes, and other organs, thus participating in the establishment of a prometastatic niche and the degradation of ECM [154]. EEVs derived from CML cells promote cell survival, adhesion, and metastasis by inducing IL-8 proinflammatory chemokine production from bone marrow stromal cells [155]. However, EEVs released from acute myelogenous leukemia (AML), which are enriched in mRNAs and miRNAs essential for AML pathogenesis, induce proliferative and angiogenic profiles when transferred to bone marrow stromal cells [132].

EEVs containing specific proteins—mRNAs, and miRNAs released from breast cancer cells [156] and melanoma [157], respectively—contribute to the formation of premetastatic niches in the lungs and lymph nodes. In addition, ECM remodeling is essential for tumor cell invasion and metastasis by influencing intercellular cross-talk between tumor and stromal cells [158]. Tumor-derived EEVs can bind to specific components of the ECM, depending on a set of EEVs adhesion molecules. For instance, high expression of *CD44* and *α_6_β_4_* mediate hyaluronic acid and laminin 332 binding, respectively, inducing degradation of ECM [134]. These EEVs are rich in matrix metalloproteinases (MMPs), MMP activators and other proteases including urokinase plasminogen activator receptor (uPAR), metallopeptidases (ADAMs), and hyaluronic acid proteases (HAdase). The EEVs content induced the degradation of ECM components and matrix proteins type I and IV collagens, laminins, and fibronectin [134]. EEVs derived from gastrointestinal stromal tumor patients show enhanced MMP1 secretion by smooth muscle cells compared to healthy donors and induce tumor cell invasion. In contrast, inhibition of EV-mediated MMP1 secretion decreases tumor invasiveness [159]. In addition, EEVs derived from metastatic head and neck squamous cell carcinoma cells are important for invadopodia formation and activity, key structures for the invasion process [160]. EEVs from GBM microenvironment were found to mediate aggressiveness and tumorigenesis. Certain EEVs were found to transport PTPRZ1–MET fusion (ZM fusion) transcript and can be internalized by recipient cells inducing GBM cell migration and invasion [161]. The role of EEVs in promoting metastasis in CRC was recently highlighted. In particular the level of EEVs miRNA (miR-25-3p, miR-130b-3p, and miR-425-5p) were correlated with the progression and metastasis of CRC [136].

### 2.4. Cancer EEVs and Drug Resistance 

EEVs are involved in the mechanisms of drug resistance and may influence the efficacy of chemotherapeutics, either by exchanging drug-resistant information or by exporting drugs from tumor cells, protecting cancer cells from drugs. Melanoma cells treated with cisplatin actively eliminate cisplatin via EEVs in a pH-dependent manner [162]. Moreover, enhanced secretion of EEVs was demonstrated after the treatment of A549 lung cancer cells with cisplatin [163]. EEVs from the MCF-7 cell line were rich in miRNAs such as miR-196a, miR-21, miR-222, miR-29a, miR-100, miR-30a [141,142], and P-glycoprotein (P-gP) [145], which are implicated in drug resistance. Moreover, adding these EEVs into recipient drug-sensitive tumor cells induces resistance to the treatment [142]. In a different study on prostate cancer cells, EEVs’ transfer of MDR-1/P-glycoprotein increased drug resistance [144]. On the other hand, EEVs can competitively bind to and inhibit a drug’s action. For instance, EEVs deriving from lymphoma cells are rich in CD20 surface markers and can compete with Rituximab, a monoclonal antibody against CD20 used in hematological cancers; therefore, they protect tumor cells from the cytotoxicity induced by rituximab-dependent complementation [164]. In addition, sensitivity to rituximab was inhibited by EEV delivery of ATP-binding cassette (ABC) transporter A3 (ABCA3), which mediated subcellular drug sequestration in acute myeloid leukemia (AML) [165]. In a similar mechanism, EEVs released by breast cancer cells overexpressing HER2 inhibited the antiproliferation activity of trastuzumab, an antibody targeting HER2 [139]. Similarly, EEVs derived from bone marrow MSC induced dormancy of breast cancer cells by delivering miR-23b, thus escaping from drug action because dormant tumor cells are usually unresponsive to traditional chemotherapies [129]. miR-223 is involved in the cross-talk between macrophages and epithelial ovarian cancer cells and could mediate chemotherapy resistance [166]. Finally, a large number of soluble factors synthesized and secreted from drug-damaged stromal cells, including CXCL1, CXCL2, GM-CSF, SPINK1, IL-6, IL-8, AREG, WNT16B, SFRP2, and TIMP1, promote the survival of recipient cells [138].

## 3. Importance of EEV Content in Cancer Diagnostics

EEV content (nucleic acids, proteins, and lipids) is attracting interest as a novel cancer biomarker for diagnosis and prognosis. These considerations are based on particular features of EEVs such as the variable contents of DNA, RNA, proteins, and lipids of tumoral origin [167]. The biochemical characteristics of the bilayer membrane of EEVs made from lipids have a protective role for EEV contents from degradative enzymes present in the blood. The small dimensions of EEVs, their permeability to plasma membranes, and a widespread presence in most body fluids suggest the use of EEVs as a liquid biopsy for diagnostic purposes [168]. Additionally, the low concentration of cancer-derived nucleic acids and proteins in the early stage of disease in a complex biological sample like blood makes it a big challenge that can be solved by the extraction of cancer-derived EEVs contained in the blood (10^9^ EEVs/mL) [169].

Since EEVs provide a barrier against enzymes and mainly RNases, a lot of research is focusing on the effective use of EEVs for diagnostic approaches based on miRNA [170]. Several studies showed the role of blood-derived EEV miRNA in the dysregulation of normal physiology, which supports their use during early cancer detection (Table 4). Mckiernan et al. demonstrated the utility of combining the expression profile of urine EEVs with standard-of-care protocols for prostate cancer patients to improve the diagnosis [171].

EEVs derived from cancer cells also contain tumor-specific proteins. Recent proteomic analyses suggested the presence of specific protein profiles shown by EEVs of cancer patients; therefore, EEVs proteins might be used as diagnostic tools for different types of cancer during the early stages. One distinctive example is the glypican-1 protein found in circulating EEVs [31]. Glypican-1 positive EEVs were detected with high specificity and sensitivity in the serum of patients in the early stages of pancreatic cancer (Table 5). Furthermore, glypican-1-positive circulating EEVs were found to be correlated with tumor burden and the overall survival in pre- and postsurgical stages. Depending on these relevant data, EEV proteins exhibit a high utility as noninvasive screening and diagnostic tools for cancer detection.

Up to now, it has been a major challenge to identify and quantify EEV proteins in body fluids due to their high heterogeneity [204]. It should be noted, for instance, that reliable and cost-effective technologies for EEV extraction from a small volume of human body fluid are not present. In addition, ultracentrifugation techniques are still used in EEV isolation in basic research but are not efficient for clinical use. As a consequence, it is important to develop advanced methods for EEV protein analysis that have sensitivity and specificity.

### 3.1. Novel Approaches for EV Detection in Clinic

Researchers reported the use of specific noninvasive technologies based on antibodies to analyze EEVs proteins from body fluids such as miniaturized nuclear magnetic resonance-based platform (μNMR) [205], protein microarray [184], flow cytometry [206], and nanoplasmonic sensing [207]. Although these methods are widely used to purify and analyze EEVs, their conversion to clinical technology is mostly impractical. For clinical study, the development of a simple and ultrasensitive technique would be useful for the isolation and molecular analysis of circulating EEVs. The field of circulating EV detection is still new, and novel technologies will have to address specific challenges such as slow analysis, low sample volumes, and body fluid complexity [208,209]. ExoScreen is an applicable example of a novel technology in that it uses circulating (both CD147- and CD9-positive) EEVs to detect colon cancer. This technology can detect specific tumoral EEVs in just 5 µL of serum sample without EV purification, using an amplified luminescent proximity homogeneous assay with photosensitizer beads. Compared with classical markers of cancer, the receiver operating curve indicated a better diagnostic characteristic of CD147/CD9 double-positive EEVs. Thus, ExoScreen showed a high impact as a new ultrasensitive technique based on the use of EEVs for liquid biopsy to detect cancer [196]. Another example is the work by Kanwar et al., in which a low-cost and simple microfluidic-based platform called “ExoChip” was developed. It was prepared in polydimethylsiloxane (PDMS) and functionalized with an anti-CD63 EEVs marker to isolate circulating extracellular vesicles enriched in EEVs directly from the blood serum. Thereafter, the extracellular vesicles are stained with a lipophilic cationic indocarbocyanine fluorescent dye (DiO) and quantified with a standard plate reader. This allows for the isolation and quantification of EEVs simultaneously in a single device. The recovered EEVs contain intact RNA, enabling the profiling of EEV miRNA through an open-array analysis with a potential application in biomarker discovery [210].

### 3.2. EEVs as Biomarkers in Clinical Trials

Several preclinical studies support clinical investigations to clarify and apply the use of EEVs as diagnostic tools for human biofluids with noninvasive procedures. Table 6 summarizes the up-to-date clinical studies (observational) using EEVs as liquid biopsies. From the recruiting studies, researchers from the University Hospital of Bordeaux in France have designed a study (NCT03032913) to analyze the diagnostic accuracy of circulating tumor cells (CTCs) and onco-EV quantification in the diagnosis of pancreatic cancer from approximately 20 patients with a recent diagnosis or suspicion of pancreatic ductal adenocarcinoma (PDAC) and 20 participants without cancer. In a second recruiting study (NCT03108677), researchers from Leo W. Jenkins Cancer Center Greenville, NC, USA performed a four-patient pilot study on blood samples and found that the RNA levels and mutational profile of circulating EEVs from patients with or without lung metastasis were significantly different. Researchers from the University of New Mexico Cancer Center (Albuquerque, NM, USA) developed a new test based on EEVs to detect Human Papillomavirus-positive proteins in the blood or saliva to improve the diagnosis of oropharyngeal squamous cell carcinoma (OPSCC) (NCT02147418). Another study at the Medical University of Nanjing in China aims to characterize EEV ncRNAs derived from naïve cholangiocarcinoma patients as effective circulating biomarkers before and after surgical resection. A combined phase 2 clinical trial (NCT03228277) at Konkuk University Medical Center of Korea was designed to assess the antitumor efficacy of Olmutinib (Olita^®^) administered to patients with T790M mutations in non-small-cell lung cancer (NSCLC). As a measure of objective response rate (ORR), the investigators planned to use DNA extracted from extracellular vesicles from bronchoalveolar lavage fluids. Starting in 2016 in the USA, novel cancer diagnostic products relying on EV profiling [211] have reached the market. ExoDx^®^ Prostate (IntelliScore) and ExoDx^®^Lung (ALK) are two test kits based on the liquid biopsy approach that are commercially available to detect prostate and lung cancer markers, respectively, in blood and urine samples.

It should be noted that not all the studies reported in Table 6 have yet published their results, although they reflect the interest in EEVs in the diagnostic field and represent a next-generation approach to early cancer detection.

## 4. EEVs as Vehicles for Drug Delivery

The ideal drug delivery system would deliver the drug only to specific locations, avoiding its recognition and degradation by the body’s immune system. Such systems would also control the release of cargo molecules upon selective stimuli. EEVs offer a new solution for unmet clinical treatments because they can act as a nanoscale drug delivery system and transport their cargo, such as proteins, DNA, and RNA [36]. Additionally, EEVs have been used to deliver interfering RNA (siRNA) [212] or pharmaceutically active substances for drug delivery such as paclitaxel [213], doxorubicin [214], and curcumin [215], as well as peptide- and protein-based therapeutics [216]. Because of their small and native structure, they are able to avoid phagocytosis; cross biological barriers, including the blood‒brain barrier (BBB) [41]; and internalize in the cell through clathrin-mediated endocytosis and micropinocytosis [217] by passing lysosomes. They also have a hydrophilic core, which makes them suitable to host soluble drugs [218] and gives them a natural targeting capacity [219]. One of the advantages of using EEVs as nanocarriers is their small size, which gives them the ability to penetrate deep into tumors and inflamed tissues [220,221]. In addition, EEVs also have a slightly negative zeta potential for long circulation [222]. The negative surface charge allows EEVs to not interact with an endothelial surface that presents the same negative charge, and the presence of “self” surface markers like CD47 will prevent them from being phagocytized by the reticuloendothelial system (RES) [223]. EEVs could be safely delivered to the patient after being isolated from their body fluids or transferred to cell cultures to be modified [224]; they possess an inherent targeting characteristic and show little long-term accumulation in any organ or tissue, so they may also have fewer off-target effects and lower toxicity [36]. Because EEVs are natural products, the immune response they induce is very minor compared to other drug delivery systems such as liposomes and virus-based drug delivery [225,226]. The cellular uptake of EEVs is facilitated compared to various synthetic methods of drug delivery [227]. The uptake and pathways in which EEVs are internalized are the result of the presence of key proteins on the EEV surface such as tetraspanins and integrins [87]. Due to these biocompatible properties, EEVs are promising nanosize drug carriers for clinical applications. 

### 4.1. Loading EEVs with Therapeutic Cargos

The loading of EEVs with therapeutic cargos is carried out via two approaches. Firstly, naïve EEVs could be purified from cell media and the drug is loaded by the free diffusion or electroporation technique [215,228]. Successful studies included the loading of small molecules such as curcumin, antioxidants [216,229], or anticancer agents such as doxorubicin (DOX) [43] and paclitaxel (PTX) [230]. Through this approach, EEV drug formulations could be obtained in large quantities and standardized to obtain similar preparations. The second approach consists of loading parental cells with a drug, then releasing the drug in EEVs. Murine SR4987 MSCs-secreted EEVs were loaded with PTX by simply incubating the parental cells with the drug. These cells produced an important quantity of PTX-loaded EEVs [213]. This technique was also used to encapsulate in EEVs several effective anticancer drugs such as Etoposide, Carboplatin, Irinotecan, Epirubicin, and Mitoxantrone. These EEVs were able to inhibit the proliferation of the human pancreatic cell line CFPAC-1, and induced immunogenicity and NK cell responses [213]. It should be noted that prolonged in vitro treatment can affect the properties of drugs as well as the bioactivity and stability of EEVs [223].

Donor cells could also be transfected with DNA-encoding therapeutic compounds that will be released in EEVs [37,231,232]. This approach succeeded in delivering chicken egg ovalbumin OVA to vesicles’ membranes [232]. Another study also revealed the importance of this process; plasmid DNA (pDNA)-encoding therapeutic protein catalase [233] or glial cell-line derived neurotrophic factor (GDNF) [231] was used to transfect macrophages for neurodegenerative disorder treatment. The association of adeno-associated virus (AAV) capsids with extracellular vesicles (EEVs) diminished their immunogenicity and improved gene delivery in vitro [234]. However, each of these approaches has advantages and limitations that may dictate the type of cargo, the site of the disease, and conditions. As described before, most cargos are loaded into EEVs by passive loading methods or electroporation. The need for multiple purification steps represents a disadvantage of these approaches, affecting the quality and the membrane integrity (in particular for electroporation) of EEVs [223].

### 4.2. Small Molecules

EEVs were exploited to deliver low-molecular-weight drugs [213,230,235]. Our group isolated EEVs from MDA-MB-231 breast and STOSE ovarian cancer cell lines and loaded with DOX through electroporation. It was demonstrated that EEVs reduced the toxicity of DOX. Physical examination and body weight analyses of mice treated with doxorubicin (exoDOX) showed a better safety profile than DOX-treated mice, in turn setting up exoDOX as a potential alternative therapy for breast and ovarian cancers [42,43].

A similar result was obtained by incorporating PTX into EEVs to treat tumors in mouse models. MSCs were found to acquire effective in vivo antitumor activity after PTX incorporation in a dose-dependent manner, able to take up the drug and later release it through their extracellular vesicles [213]. The authors also reported an optimized formulation of PTX-loaded EEVs by incorporating an aminoethyl anisamide-polyethylene glycol (AA-PEG) vector moiety to target the sigma receptor, which is overexpressed by lung cancer cells. This platform (AA-PEG-exoPTX) possesses a high loading capacity and ability to accumulate in cancer cells after systemic administration to improve therapeutic outcomes [236].

Vesicles were also utilized in phototherapy. By comparing membrane vesicles loaded with hydrophobic photosensitizers to polymer-based synthetic nanoparticles, a superior phototherapeutic effect was obtained in membrane vesicles; they fuse more effectively with cancer cell membranes. These liposomal-mediated engineered microvesicles allowed hydrophobic photosensitizers to penetrate spheroids and in vivo tumors to enhance the therapeutic efficacy [237].

In another study, EEVs loaded with curcumin, an anti-inflammatory small molecule compound, improved its solubility, circulation time, and delivery to the brain without altering the drug’s therapeutic activity. Curcumin was able to protect mice from lipopolysaccharide-induced brain inflammation [215,216]. EEVs are also used for different therapeutic purposes, such as delivering exogenous siRNA [41,224,238,239].

### 4.3. Nucleic Acids

EEVs and extracellular vesicles naturally deliver mitochondrial and genomic DNA, mRNA, miRNA, and various noncoding RNAs [240]. As they are already nanocarriers, they were suggested as carriers for nucleic acids. The incorporation of this genetic material could be achieved by electroporation, such as electroporating siRNA into DC-derived EEVs [41] or using sonication [241]. Electroporation is used to enhance the permeability of the membrane in order to facilitate the loading of nucleic acids inside the EEVs [41,242,243,244]. Moreover there are some limitations to this technique, such as extensive siRNA aggregation, which may cause inaccurate estimation of the amount of siRNA loaded into EEVs [243].

EEV vesicles can deliver siRNA to human blood cells. For example, plasma EEVs were used as a gene delivery vector to transport administered siRNAs to human monocytes and lymphocyte blood cells, selectively silencing the mitogen-activated protein kinase [224]. Moreover, it was demonstrated that EEVs can efficiently deliver siRNA against RAD51 and RAD52 to recipient cells in vitro, which play an important role in homologous strand exchange, a key step in DNA repair through homologous recombination [239,245]. These siRNAs caused post-transcriptional gene silencing in target cells. Specifically, the siRNA delivered against RAD51 caused massive cancer cell death [239]. During tumor development, tumor-associated macrophages secrete angiogenic factors such as VEGF to promote tumorigenesis. The oncomir miR-150 targets tumor-associated macrophages that increased the secretion of VEGF. In vivo angiogenesis was attenuated by cell-derived vesicles, delivering antisense RNA to miR-150 into mice [246].

EEVs derived from normal fibroblast-like mesenchymal cells carrying siRNA or shRNA that target oncogenic *Kras^G12D^* successfully suppressed the growth of pancreatic cancer in different mouse models [247]. Thus, human EEVs can be used as vectors for gene delivery to provide cell therapeutic siRNAs, overcoming the obstacle of crossing the cellular plasma membrane that limits RNA interference-based therapeutics.

### 4.4. Proteins

Among the engineered nanoparticles, cell-derived EEVs have recently been emphasized as a promising therapeutic strategy for in vivo and in vitro delivery of proteins [248]. Therapeutic proteins could be loaded into EEVs by overexpression in donor cells. To preserve the carried therapeutic proteins against degradation in host cells and increase the EEV loading capacity, a polymer-based nanocontainer could be used before the loading, which ensures low cytotoxicity. For example, macrophage-carried nanoformulated catalase was internalized by endocytosis, then trafficked to recycling endosomes. Catalase was subsequently released in EEVs and transferred from macrophages to adjacent cells [249].

In addition to chemotherapeutic drugs, EEVs can deliver proteins in order to target cancer cells. It is demonstrated that EEVs loaded with the protein Survivin-T34A induce apoptosis in pancreatic adenocarcinoma cell lines and enhance cell sensitivity to gemcitabine [250]. In 2017, Koh et al. engineered EEVs harboring signal-regulatory protein alfa variants (SIRPα), a membrane protein expressed on the surface of phagocytes. By interacting with CD47, a “don’t eat me” protein, the SIRPα‒CD47 complex blocks the phagocytosis of tumor cells. Exploiting this mechanism, SIRPα competitively inhibits the CD47 protein on tumor cells. This strategy enhances the capacity of phagocyte toward tumor cells in the presence of engineered EEVs both in vitro and in vivo. Mice tumor xenograft treated with SIRPα-EEVs showed a reduction in tumor volume compared to untreated mice [250]. Based on this principle, another research group engineered EEVs derived from M1 macrophages with anti-SIRPα and -CD47 antibodies through a pH-sensitive linker. In the tumor microenvironment, where pH is decreased by the fast and anabolic metabolism of tumor cells, linkers are cleaved and anti-CD47 and -SIRPα antibodies are released and blocked, respectively, SIRPα on macrophages and CD47 on tumor cells improve the phagocytosis of macrophages towards tumor cells. At the same time, the native M1 EEVs effectively reprogram the macrophages from protumoral M2 to antitumoral M1 [251].

## 5. Therapeutic EEVs in Clinic

The use of EEVs as a drug delivery system (DDS) reached clinical trials with a phase I clinical study in 2005, in which Escudier and his research group tested the feasibility of autologous dendritic cell (DC)-derived EEVs (DEX) pulsed with the melanoma associated antigen 3 (MAGE 3) as vaccination of metastatic melanoma patients [252] (Table 7). The secreted EEVs transferred MHC class I/peptide complexes to antigen-naïve DCs in the lymph nodes, leading to the stimulation of CD4^+^ and CD8^+^ T cells [30,253]. Fifteen patients participated in this clinical study and were treated with EEVs vaccination once per week for four weeks. No sign of grade II toxicity was reported and the maximum tolerated dose (MTD) was not achieved. Based on the results, patients with non-small-cell lung cancer (NSCLC) were treated with T autologous DEX loaded with MAGE [254]. This clinical study was carried out on a group of 13 patients (median age: 62 years old) with unresectable pretreated stage III or IV NSCLC with tumor expression of MAGE. In order to generate DCs, patients underwent leukapheresis and then DEX were produced, loaded with MAGE, and administered at weekly intervals. During the study, three DEX formulations were evaluated; all of them were tolerated with only grade 1‒2 adverse effects (flu-like illness (*N* = 1), peripheral arm pain (*N* = 1), and injection site reactions (*N* = 8)). The time between the first dose of DEX and disease progression was 30 to 429 days. Disease progression was shown in three patients before the first dose of DEX. Additionally, the patient’s survival ranged from 52 to 665 days after the first dose of DEX. Thus, the vaccination with DEX was considered functional and advanced NSCLC patients showed good tolerance for DEX therapy (NCT01159288). In 2008, another phase I study was carried by Dai et al., based on the use of EEVs vaccination, in which ascites-derived EEVs (Aex) combined with granulocyte-macrophage colony stimulating factor (GM-CSF) were used in the immunotherapy of colorectal cancer (CRC) [255]. A total of 40 patients diagnosed with advanced CRC were enrolled in this study and assigned randomly to two groups: the first treated with Aex and the second with Aex plus GM-CSF. A total of four injections were administrated once per week subcutaneously. Both therapies were found to be safe to use and well tolerated. Furthermore, Aex + GM-CSF had a beneficial role in the induction of antitumor-specific cytotoxic T lymphocyte (CTL) response.

Previous research works demonstrated the anti-inflammatory characteristics of curcumin and its delivery using EEVs as natural carriers for the treatment of brain diseases and colon cancer [216,229,256,257]. These data are the basis of a phase I clinical trial (NCT01294072) in which investigators plan to study the ability of plant EEVs to improve curcumin delivery to colon tumors. At the end of this clinical study, the effect of curcumin on the phospholipidic profile, immune modulation, and cellular metabolism will be analyzed in normal and cancer patients. The results of this clinical trial are not yet published.

It is worth mentioning that most of the initial clinical trials are ongoing and the use of extracellular vesicles for therapeutic or drug delivery applications and immunomodulatory therapies still requires large-scale research.

## 6. Conclusions

Nanomedicine has gained an important role in the treatment and diagnosis of a variety of human diseases, especially cancer. Nanoparticles have been shown to possess suitable properties such as improved pharmacokinetics, bioavailability, and passive targeting, owing to the enhanced permeability and retention effect. Besides the cited pharmacological properties, the nanoformulation also improved the physicochemical properties of drugs and could be engineered in order to modify their surface for active targeting [258,259,260]. Several nanoparticle formulations, such as lipids and polymer-based materials, have already been approved and are in use [20,261,262]. Although such nanocarriers show good pharmacokinetic and safety profiles, together with enhanced bioavailability, the use of naturally occurring EEVs could be of help in overcoming the off-target effects of lipid- and polymer-based nanocarriers since EEVs are naturally covered with several biomolecules useful for tumor targeting [263,264]. In addition, the targeting ability could be improved by molecular engineering [41]. A combination of EEVs with nanoparticles is also possible to combine the positive characteristics of both nanomaterials [265]. Different from the targeting abilities, several challenges should be considered for enhancing EEVs as drug vehicles, such as the production of reproducible EEVs, the identification of the best donor cells for optimal EEVs production, and an efficient loading system. Optimization in order to avoid immunogenic responses and ensure correct retention time in particular tissues is also challenging.

More information is needed to modify EEVs as biologically active carriers for different pathologies [226,266,267,268,269]. The goal of these challenges will serve as an essential stage for the industrial development of EEVs as therapeutic vehicles, including quality control, scaling up production, toxicological and pharmacokinetic analyses, and, finally, clinical trials.

In the field of early cancer detection, EEVs have also attracted growing interest due to their tissue- or disease-specific content [270]. Nucleic acids, which circulate in the blood and can be protected by EEVs from degradation, represent a unique population of biomarkers. Of course, the lack of knowledge about EV generation, purification, and biobank conservation are still major limitations on their application in diagnosis. More effort should be made in these fields to generate solid data and allow for rapid translation to patients. At that point, next-generation sequencing technologies of serum EEVs DNA and RNA influence early cancer diagnosis. Utilizing multiomics approaches to EEVs will provide a step forward in the understanding of cancer [271].

## Figures and Tables

**Table 1:** Main EEV membrane components.

Proteins	Function	References
Alix	MVB formation	[46,61,62,63]
Gag		
TSG101		
Hsp70, Hsp84, Hsp90	Protein‒protein interaction	
Advillin	Cytoskeletal Proteins	
Actin, Ezrin		
CAP1, Talin		
Radixin		
Vimentin		
ATPase	Enzymes	
ATP Citrate lyase		
Asp amino-transferase		
a-enolase		
G6P Isomerase		
Peroxiredoxin 1		
Cholesterol	Lipid Raft	
Flotilin-1		
LBPA		
Stomatin		
CD9, CD37, CD53, CD63, CD81, CD82	Tetraspanin	
MHC I/II	Antigen presenting	
CD86		
AP-1, Arp2/3, SNAP	Membrane fusion and transport	
Dynamin		
RabGD1, Rab5,7, Rap1B		
Syntaxin		
CD11a, b, c	Adhesion molecules	
CD146, CD166, CD326		
ALCAM, ICAM-1		
CD53, LFA3		
Catenin, LCK	Signal transduction	
Erk2, FRL, Fyn, Gi2a, Gi3a, Gsa		
RhoA, C, GDI		
Sh2 phosphatase		
Syntenin, CBL		
CD18, CD55, CD59, CD147	Other proteins	
Complement factor 3		
Clathrin	Other proteins	[46,61,62,63]
Histone 1, 2, 3		
MVP, CD26, CD13		
P-selection		
peroxidase		
Thioredoxine		

**Table 2:** EEV isolation methods.

Method of EEV Isolation	Isolation Principle	Advantages	Disadvantages
Ultracentrifugation	Ultracentrifugation is based on the separation and purification of different EVs according to the size and density of the EVs applying high centrifugal speed	Reduced contamination risk Large sample capacity and yields, large extraction of EEVs	High equipment cost, time-consuming, labor-intensive, and low portability (not available at point of care). High-speed centrifugation with risk of EEV damage and lack of methods for the precise assessment of EEV damage
Density-gradient centrifugation	Separation of vesicles according to their density	High EEV purification	EEVs could be divided into multiple subsamples Subject to operator-dependent variability
Size-exclusion chromatography	Based on the size difference between EEVs and other constituents	Ultrafiltration: Fast, no special equipment needed, good portability, possibility of direct RNA extraction, and low equipment cost SEC: high-purity EEVs, gravity flow preserves the integrity and biological activity, moderate sample capacity, high reproducibility	Ultrafiltration: moderate purity of EEVs, risk of deterioration, possibility of elution blockage and vesicle trapping, EEV loss due to membrane attachment SEC: needs dedicated equipment and a long running time.
Polymer precipitation	Altering the solubility or dispersibility of EEVs by the use of water-excluding polymers	Relatively easy to use and does not require a specialized equipment or a long run time.	Coprecipitation of non-vesicular contaminants such as lipoproteins, as well as polymers; Pre- and post-isolation steps can overcome this limitation, pre-isolation typically to remove subcellular particles such as lipoproteins through centrifugation; Post-isolation to remove the polymer, using a specific (Sephadex G-25) column.
Microfluidics-based techniques	Microscale isolation based on EEV characteristics such as immunoaffinity, size, and density	Small volume of starting material Highly pure EEV isolation Not time-consuming	No standardization and large-scale tests on clinical samples No method validation Low sample capacity
Immunoaffinity chromatography	EEV capture based on specific interaction between membrane-bound antigens (receptors) of EEVs with immobilized antibodies (ligands)	Excellent for the isolation of specific EEVs. Highly purified EEVs compared with other techniques, high possibility of subtyping	High-cost reagents, low yield, cell-free samples conditions Tumor cells could inhibit immune recognition. Risk of blockage, or masking of antigenic epitopes.

**Table 3:** EEVs in cancer.

EEVs Proteins/mRNA, miRNA	Effects on Stromal Cells	Role in Tumor Malignancy	References
Carbonic anhydrase 9 (CA9)	Induced by hypoxia-inducible factor 1 (HIF1) in response to hypoxia	Angiogenesis	[119]
Cell adhesion molecule 1	Endothelial cell motility		[107,120]
FGF2 mRNA	Vessel formation		[121]
Interleukin-8	• Endothelial cell motility		[107,119,120]
	• Enhanced tumor survival		
miR-126	Formation of vessel-like endothelial structures		[122]
miR-130a	Formation of vessel-like endothelial structures		[122]
miR-155	c-MYB inhibition and vascular endothelial growth factor (VEGF) induction: promoting cell growth, metastasis, and tube formation of vascular cells		[123]
miR-210	-Secreted under hypoxic conditions -Angiogenesis induction		[124]
MMP-2 mRNA	Vessel formation		[121]
MMP-9 mRNA	Vessel formation		[121]
TGF-ß	Differentiation of fibroblasts into myofibroblast FGF2 induction (fibroblast growth factor 2) and tumor vascularization	Angiogenesis	[125]
Tspan8‒CD49d complex	VEGF-independent regulation of angiogenesis-related genes Endothelial cell proliferation, migration, and progenitor maturation		[126]
Vascular cell adhesion molecule 1	Endothelial cell motility		[107,120]
VEGF mRNA	Vessel formation		[121]
Apolipoprotein E	Migration through the transfer from cancer-associated macrophages to cancer cells	Metastasis	[114]
CXCR4	Angiogenesis and metastasis		[127]
IRF-2	VEGF-C secretion, with consequential lymphangiogenesis and metastasis		[128]
miR-23b	Dormancy induction		[129]
miR-223	Invasiveness of breast cancer cells in vitro		[130]
miR-1246	Reprogrammed macrophages to a cancer-associated phenotype with high expression of TGF-β, increasing tumor growth		[131]
Insulin-like growth factor I receptor	Activation of insulin-like growth factor I receptor signaling in bone marrow stromal cells	Premetastatic niche formation	[132]
Oncoprotein receptor tyrosine kinase (MET)	Vascular leakiness		[133]
ADAMs (metallopeptidases domain)	Matrix degradation	Premetastatic niche formation and ECM degradation	[134]
Amphiregulin (AREG)	Contributes to therapeutic resistance in tumor microenvironment		[135]
HAdase (Hyaluronic acid proteases)	Degradation of ECM components and proliferation of stromal cells		[134]
miR-25-3p, miR-130b-3p and miR-425-5p	Serum level of EEV miRNA is correlated with progression and metastasis of CRC		[136]
uPAR (urokinase plasminogen activator receptor)	Degradation of the ECM of stroma lines and endothelial cells (EC)		[134]
ABC (ATP binding cassette) transporter A3	Subcellular drug sequestration	Therapy resistance	[137]
CXCL1, CXCL2 (Chemokine ligand)	Tumor survival		[138]
GM-CSF (colony-stimulating factor	Tumor survival		[138]
HAdase (Hyaluronic acid proteases)	Degradation of ECM components and proliferation of stromal cells		[134]
HER2 (human epidermal growth factor receptor)	Inhibition of the antiproliferation activity of trastuzumab in breast cancer		[139]
IL-6	Drug sensitivity		[140]
miR-21	Increase of docetaxel resistance in MCF-7 cells and recipient cells		[141]
miR-29a	Increase of docetaxel resistance in MCF-7 cells and recipient cells		[141]
miR-30	Increase of docetaxel resistance in MCF-7 cells and recipient cells		[142]
miR-100	Increase of docetaxel resistance in MCF-7 cells and recipient cells		[142]
miR-122	Reduction of drug susceptibility in chemosensitive cells in CRC		[143]
miR-196a	Increase of docetaxel resistance in MCF-7 cells and recipient cells		[141]
miR-222	Increase in docetaxel resistance in MCF-7 cells and recipient cells		[142]
Multidrug-resistance transport (MDR-1)	Drug resistance	Therapy resistance	[144]
P-glycoprotein (P-gP)	Drug resistance		[145]
SFRP2	Wnt pathway modulator, produced by human primary fibroblasts after genotoxic treatments		[146]
TIMP1 (metallopeptidase inhibitor 1)	Cell proliferation and tumor survival		[138]

**Table 4:** EEVs miRNAs in cancer.

Tumor	Body Fluids	Biomarkers	Isolation/Detection Method	References
Breast cancer	Plasma	miR-200a, miR-200c, miR-205	Ultracentrifugation/nest-qPCR	[172]
Cervical cancer	Cervicovaginal lavage	miR-21, miR-146a	Ultracentrifugation/RT-PCR	[173]
Colorectal cancer	Serum	Let-7a, miR-1229, miR-1246, miR-150, miR-21, miR-223, miR-23a	Step-wise centrifugation/ultracentrifugation/miRNA microarray, qRT-PCR	[174]
Glioblastoma	Serum	miR-320, miR-574-3p	Exoquick/RT-PCR	[175]
	Cerebrospinal fluid	miR-21	Ultracentrifugation/qRT-PCR	[176]
Lung cancer	Plasma	miR-151a-5p, miR-30a-3p, miR-200b-5p, miR-629, miR-100, miR-154-3p	ExoQuick/qRT-PCR	[177]
	Plasma	Let-7f, miR-30e-3p, miR-223, miR-301	Ultracentrifugation/RT-PCR	[178]
Ovarian cancer	Serum	miR-21, miR-141, miR-200a, miR-200b, miR-200c, miR-203, miR-205, miR-214	Modified MACS procedure with anti-EpCAM/miRNA microarray	[73]
Pancreatic cancer	Serum	miR-17-5p, miR-21	Ultracentrifugation/RT-PCR	[179]
	Serum	miR-1246, miR-4644, miR-3976, miR-4306	Ultracentrifugation/qRT-PCR	[180]
Prostate cancer	Serum	miR-141, miR-375	ExoMir/RT-PCR	[181]
	Urine	miR-107, miR-574-3p		
		δ-catenin, PCA-3, TMPRSS2:ERG	Ultracentrifugation/nested PCR	[182]

**Table 5:** Cancer EEVs protein content.

Tumor	Body Fluids	Biomarkers	Isolation/Detection Method	References
Spongioblastoma	Serum	EGFR-VIII	Ultracentrifugation	[183]
	Plasma	EGFR, PDPN, IDH1	Differential centrifugation, filtration followed by ultracentrifugation/ELISA	[184]
Nasopharyngeal cancer	Plasma	Galactin-9	Ultracentrifugation/Immunomagnetic capture	[185]
	Serum, saliva	LMP1, BARF1	Ultracentrifugation and sucrose gradient/Immunoprecipitation	[186]
Melanoma	Plasma	TYRP2, VLA-4, Hsp70, Hsp90, CD63, caveolin-1	Ultracentrifugation/ELISA	[74,133]
	Serum	MRD-9, GFP78	Polymer precipitation/Immunoblotting	[187]
Breast cancer	Plasma	CEA, tumor antigen15-3	Differential centrifugation/Immuno-enzymometric assay, latex-amplified immuno-turbidimetry-	[188]
	Serum	Survivin, Survivin-2B	Polymer precipitation/ELISA	[189]
Lung cancer	Urine	Leucine-rich a-2glycoprotein (LRG1)	Ultracentrifugation/1-D SDS-PAGE	[190]
	Serum	CD9-CD91, EGFR, EpCAM, CEA	Porous monolithic silica microtips/Mass Spectrometry and ELISA, Extracellular Vesicle Array	[191,192,193]
Pancreatic cancer	Serum	Glypican-1, CD44v6, Tspan8, EpCam, MET, CD104, MIF	Ultracentrifugation/Mass Spectrometry	[31,180,194]
Gastric cancer	Plasma	HER-2/neu, CCR6	Ultracentrifugation/FACS (fluorescence-activated cell sorting)	[195]
Colorectal cancer	Serum	CD147, CD9	ExoScreen using photosensitizer-beads	[196]
Ovarian cancer	Serum	EpCAM	Modified MACS (magnetic-activated cell sorting) procedure with anti-EpCAM	[73]
Ovarian cancer	Plasma	Claudin-4, TGFβ1, MAGE3/6, CD24, L1CAM, ADAM10, EMMPRIN	Sucrose gradient separation/Immunoblotting	[197,198,199,200]
Prostate cancer	Plasma, serum	Survivin	Ultracentrifugation/ELISA	[201]
Bladder Cancer	Urine	TACSTD2	Ultracentrifugation/liquid chromatography-tandem mass spectrometry	[202]
Renal cell carcinoma	Urine	MMP-9, EMMPRIN, Carbonic anhydrase	Differential centrifugation/liquid chromatography-tandem mass spectrometry	[203]

**Table 6:** EEV clinical trials in cancer diagnosis.

Title of the Study	Start Date	Location	Condition	Description	Interventions	Status	Clinical Trial Identifier
Identification and Characterization of Predictive Factors of Onset of Bone Metastases in Cancer Patients	3 December 2018	Palermo, Italy	Bone Metastases	Step 1: assess changes in miRNAs and protein content of circulating tumor EEVs Step 2: establish the relationship between plasma levels of circulating tumor EEVs and their content in miRNAs with overall survival and progression of the disease	Blood	Recruiting	NCT01668849
Prostasomes as Diagnostic Tool for Prostate Cancer Detection	3 October 2018	Hackensack, NJ, USA	Prostate cancer	Step 1: a validation phase where the purification of prostasomes will be tested on plasma collected from prostate cancer patients Step 2: a molecular testing phase where the contents of the purified prostasomes will be evaluated on their ability to determine the grade of the prostate tumor	Blood	Recruiting	NCT03694483
Interrogation of EEV-mediated Intercellular Signaling in Patients with Pancreatic Cancer	19 March 2015	NY, USA	Pancreatic Cancer	To isolate and analyze EEVs, which are tiny carriers of important proteins, and nucleic acids that serve as messenger systems in the blood and tissue. Blood and tissue from patients with pancreatic cancer will be compared with blood and tissue from patients with noncancerous pancreatic disease. Patients without cancer will allow the investigators to establish “normal” values, which currently do not exist. The investigators will then look to see whether EEVs’ activity has a connection to disease recurrence and outcomes in patients.	blood and tissue	Active, not recruiting	NCT02393703
			Benign Pancreatic Disease				
Diagnostic accuracy of circulating tumor cells (CTCs) and Onco-EEVs quantification in the diagnosis of pancreatic cancer (PANC-CTC)	26 January 2017	Bordeaux, France	Pancreatic ductal adenocarcinoma (PDAC)	Step 1: test three CTCs isolation methods and analyze by flow cytometry the presence of onco-EEVs in a culture media of pancreatic cell lines Step 2: examine the diagnostic accuracy of these blood tumor elements (CTCs and EEVs) for the diagnosis of cancer of patients with PDAC suspicion or recent diagnosis and their value for disease monitoring	Blood/ Portal vein blood samples	Recruiting	NCT03032913
EEV testing as a screening modality for human Papillomavirus-positive oropharyngeal squamous cell carcinoma	26 May 2017	NM, USA	Oropharyngeal Cancer	To develop a new test that can detect certain HPV proteins in the blood or saliva to help improve detection of OPSCC	N/A	Recruiting	NCT02147418
Clinical research for the consistency analysis of PD-L1 in cancer tissue and plasma EEVs	7 September 2016	Xinqiao Hospital of Chongqing, China	Non-small-cell lung cancer (NSCLC)	To explore the consistency analysis of PD-L1 expression level detected in cancer tissues and plasma EEVs, guiding clinical practice of radiotherapy combined with immunotherapy	Liquid biopsy	Not yet recruiting	NCT02890849
Clinical research for the consistency analysis of PD-L1 in lung cancer tissue and plasma EEVs before and after radiotherapy	17 August 2016	Xinqiao Hospital of Chongqing, China	Non-small-cell lung cancer (NSCLC)	To explore the consistency analysis of PD-L1 expression level detected in tissues and plasma EEVs before and after radiotherapy	Radiotherapy	Not yet recruiting	NCT02869685
Study of molecular mechanisms implicated in the pathogenesis of melanoma role of EEVs	4 December 2016	Nice, France	Metastatic Melanoma	To study the effect of EEVs produced by senescent melanoma cells in the development and progression of melanoma in vitro and in vivo using cell cultures and animal models	Blood test	Unknown	NCT02310451
Pilot prognostic study via urine EEVs biological markers in thyroid cancer patients	11 August 2016	National Taiwan University Hospital, Taiwan	Thyroid Cancer	To detect the prognostic markers in order to find new therapeutic mechanisms and medications for patients with poorly differentiated or anaplastic thyroid cancer	N/A	Not yet recruiting	NCT02862470
Olmutinib trial in T790M (+) NSCLC patients detected by liquid biopsy using BALF extracellular vesicular DNA	24 July 2017	Seoul, Korea	Non-small-cell lung cancer (NSCLC)	To evaluate the efficacy of Olmutinib (Olita^®^) in patients with T790M-positive non-small-cell lung cancer (NSCLC), confirmed using DNA extracted from extracellular vesicles of bronchoalveolar lavage fluid	Treatment with Olmutinib	Recruiting (Phase 2)	NCT03228277
ncRNAs in EEVs of cholangiocarcinoma	5 April 2017	Jiangsu, China	Cholangiocarcinoma	To characterize the ncRNAs of cholangiocarcinoma-derived EEVs: Step 1: see if this EEV biomarker is a useful diagnostic tool. Step 2: evaluate the prognostic and predictive values of cholangiocarcinoma EEV levels in plasma in a prospectively recruited cohort of cholangiocarcinoma patients before and after surgical resection	N/A	Recruiting	NCT03102268
			Benign Biliary Structure				

**Table 7:** EEV clinical trials for new cancer treatments.

Name	Formulation	Bioactive Compound	Company	Admin. Route	Size	Target	Description	Conclusions	Clinical Trial Identifier (Phase Status)
INF-γ-Dex	Tumor antigen-loaded dendritic cell derived EEVs	MHC class I- and class II- restricted cancer antigens and INF-γ	Gustave Roussy, Cancer Campus, Grand Paris	Vaccination (intravenous)	100 nm	NSCLC	To boost NK and T cell immune responses in NSCLC as maintenance immunotherapy after chemotherapy. Primary outcome: at least 50% of patients with PFS at four months after chemotherapy cessation	Dex stimulated NK cells but no induction of T cells was seen in patients. Primary endpoint was not reached	NCT01159288 (Phase II)
GELNs	Grape EEV-like nanoparticles	Curcumin	James Graham Brown Cancer Center	Oral	400 nm (out of canonic range)	Colon cancer	To estimate the effect of a fixed concentration of curcumin when delivered by plant EEVs compared to oral tablets of curcumin alone in CRC patients. Primary outcome: to evaluate the concentration of curcumin in normal and cancerous tissues	N/A	NCT01294072 (Phase I)

## References

[1] Mignot G., Roux S., Thery C., Ségura E., Zitvogel L. (2006). Prospects for exosomes in immunotherapy of cancer. J. Cell. Mol. Med..

[2] Johnstone R.M., Bianchini A., Teng K. (1989). Reticulocyte maturation and exosome release: Transferrin receptor containing exosomes shows multiple plasma membrane functions. Blood.

[3] Chaput N. (2006). Dendritic cell derived-exosomes: Biology and clinical implementations. J. Leukoc. Biol..

[4] Blanchard N., Lankar D., Faure F., Regnault A., Dumont C., Raposo G., Hivroz C. (2002). TCR activation of human T cells induces the production of exosomes bearing the TCR/CD3/zeta complex. J. Immunol..

[5] Heijnen H.F., Schiel A.E., Fijnheer R., Geuze H.J., Sixma J.J. (1999). Activated platelets release two types of membrane vesicles: Microvesicles by surface shedding and exosomes derived from exocytosis of multivesicular bodies and alpha-granules. Blood.

[6] Raposo G., Nijman H.W., Stoorvogel W., Leijendekker R., Harding C.V., Melief C.J.M., Geuze H.J. (1996). B lymphocytes secrete antigen-presenting vesicles. J. Exp. Med..

[7] Zitvogel L., Regnault A., Lozier A., Wolfers J., Flament C., Tenza D., Ricciardi-Castagnoli P., Raposo G., Amigorena S. (1998). Eradication of established murine tumors using a novel cell-free vaccine: Dendritic cell-derived exosomes. Nat. Med..

[8] Laulagnier K., Motta C., Hamdi S., Roy S., Fauvelle F., Pageaux J.F., Kobayashi T., Salles J.P., Perret B., Bonnerot C. (2004). Mast cell- and dendritic cell-derived display a specific lipid composition and an unusual membrane organization. Biochem. J..

[9] Schorey J.S., Bhatnagar S. (2008). Exosome function: From tumor immunology to pathogen biology. Traffic.

[10] Nguyen D.G., Booth A., Gould S.J., Hildreth J.E.K. (2003). Evidence that HIV budding in primary macrophages occurs through the exosome release pathway. J. Biol. Chem..

[11] Fader C.M., Savina A., Sánchez D., Colombo M.I. (2005). Exosome secretion and red cell maturation: Exploring molecular components involved in the docking and fusion of multivesicular bodies in K562 cells. Blood Cells Mol. Dis..

[12] Fauré J., Lachenal G., Court M., Hirrlinger J., Chatellard-Causse C., Blot B., Grange J., Schoehn G., Goldberg Y., Boyer V. (2006). Exosomes are released by cultured cortical neurones. Mol. Cell. Neurosci..

[13] Ge R., Tan E., Sharghi-Namini S., Asada H.H. (2012). Exosomes in Cancer Microenvironment and Beyond: Have we Overlooked these Extracellular Messengers?. Cancer Microenviron..

[14] Van Niel G., Heyman M. (2002). The epithelial cell cytoskeleton and intracellular trafficking. II. Intestinal epithelial cell exosomes: Perspectives on their structure and function. Am. J. Physiol. Gastrointest. Liver Physiol..

[15] Van Niel G., Mallegol J., Bevilacqua C., Candalh C., Brugière S., Tomaskovic-Crook E., Heath J.K., Cerf-Bensussan N., Heyman M. (2003). Intestinal epithelial exosomes carry MHC class II/peptides able to inform the immune system in mice. Gut.

[16] Mondal A., Tapader R., Chatterjee N.S., Ghosh A., Sinha R., Koley H., Saha D.R., Chakrabarti M.K., Wai S.N., Pal A. (2016). Cytotoxic and Inflammatory Responses Induced by Outer Membrane Vesicle-Associated Biologically Active Proteases from Vibrio cholerae. Infect. Immun..

[17] Taylor D.D., Gerçel-Taylor C. (2005). Tumour-derived exosomes and their role in cancer-associated T-cell signalling defects. Br. J. Cancer.

[18] Wolfers J., Lozier A., Raposo G., Regnault A., Théry C., Masurier C., Flament C., Pouzieux S., Faure F., Tursz T. (2001). Tumor-derived exosomes are a source of shared tumor rejection antigens for CTL cross-priming. Nat. Med..

[19] André F., Schartz N.E.C., Chaput N., Flament C., Raposo G., Amigorena S., Angevin E., Zitvogel L. (2002). Tumor-derived exosomes: A new source of tumor rejection antigens. Vaccine.

[20] Andre F., Schartz N.E.C., Movassagh M., Flament C., Pautier P., Morice P., Pomel C., Lhomme C., Escudier B., Le Chevalier T. (2002). Malignant effusions and immunogenic tumour-derived exosomes. Lancet.

[21] Cappello F., Logozzi M., Campanella C., Bavisotto C.C., Marcilla A., Properzi F., Fais S. (2017). Exosome levels in human body fluids: A tumor marker by themselves?. Eur. J. Pharm. Sci..

[22] Vidal M., Mangeat P., Hoekstra D. (1997). Aggregation reroutes molecules from a recycling to a vesicle-mediated Secretion pathway during reticulocyte maturation. J. Cell Sci..

[23] Théry C. (2011). Exosomes: Secreted vesicles and intercellular communications. F1000 Biol. Rep..

[24] Van Der Pol E., Bo A.N. (2012). Classification, Functions, and Clinical Relevance of Extracellular Vesicles. Pharmacol. Rev..

[25] Mathivanan S., Simpson R.J. (2009). ExoCarta: A compendium of exosomal proteins and RNA. Proteomics.

[26] Zomer A., Vendrig T., Hopmans E.S., van Eijndhoven M., Middeldorp J.M., Pegtel D.M. (2010). Exosomes. Commun. Integr. Biol..

[27] Valadi H., Ekström K., Bossios A., Sjöstrand M., Lee J.J., Lötvall J.O. (2007). Exosome-mediated transfer of mRNAs and microRNAs is a novel mechanism of genetic exchange between cells. Nat. Cell Biol..

[28] Batrakova E.V., Kim M.S. (2015). Using exosomes, naturally-equipped nanocarriers, for drug delivery. J. Control. Release.

[29] Keller S., Sanderson M.P., Stoeck A., Altevogt P. (2006). Exosomes: From biogenesis and secretion to biological function. Immunol. Lett..

[30] Théry C., Zitvogel L., Amigorena S. (2002). Exosomes: Composition, biogenesis and function. Nat. Rev. Immunol..

[31] Melo S.A., Luecke L.B., Kahlert C., Fernandez A.F., Gammon S.T., Kaye J., LeBleu V.S., Mittendorf E.A., Weitz J., Rahbari N. (2015). Glypican-1 identifies cancer exosomes and detects early pancreatic cancer. Nature.

[32] Li Y., Zheng Q., Bao C., Li S., Guo W., Zhao J., Chen D., Gu J., He X., Huang S. (2015). Circular RNA is enriched and stable in exosomes: A promising biomarker for cancer diagnosis. Cell Res..

[33] Tang M.K.S., Wong A.S.T. (2015). Exosomes: Emerging biomarkers and targets for ovarian cancer. Cancer Lett..

[34] Yoo J.-W., Irvine D.J., Discher D.E., Mitragotri S. (2011). Bio-inspired, bioengineered and biomimetic drug delivery carriers. Nat. Rev. Drug Discov..

[35] Haque M.E., McIntosh T.J., Lentz B.R. (2001). Influence of lipid composition on physical properties and peg-mediated fusion of curved and uncurved model membrane vesicles: “nature’s own” fusogenic lipid bilayer. Biochemistry.

[36] Kooijmans S.A.A., Vader P., van Dommelen S.M., van Solinge W.W., Schiffelers R.M. (2012). Exosome mimetics: A novel class of drug delivery systems. Int. J. Nanomed..

[37] Marcus M.E., Leonard J.N. (2013). FedExosomes: Engineering Therapeutic Biological Nanoparticles that Truly Deliver. Pharmaceuticals.

[38] Landesman-Milo D., Peer D. (2012). Altering the immune response with lipid-based nanoparticles. J. Control. Release.

[39] Katakowski M., Buller B., Zheng X., Lu Y., Rogers T., Osobamiro O., Shu W., Jiang F., Chopp M. (2013). Exosomes from marrow stromal cells expressing miR-146b inhibit glioma growth. Cancer Lett..

[40] Takahashi K., Yan I.K., Kogure T., Haga H., Patel T. (2014). Extracellular vesicle-mediated transfer of long non-coding RNA ROR modulates chemosensitivity in human hepatocellular cancer. FEBS Open Bio.

[41] Alvarez-Erviti L., Seow Y., Yin H., Betts C., Lakhal S., Wood M.J.A. (2011). Delivery of siRNA to the mouse brain by systemic injection of targeted exosomes. Nat. Biotechnol..

[42] Toffoli G., Hadla M., Corona G., Caligiuri I., Palazzolo S., Semeraro S., Gamini A., Canzonieri V., Rizzolio F. (2015). Exosomal doxorubicin reduces the cardiac toxicity of doxorubicin. Nanomedicine.

[43] Hadla M., Palazzolo S., Corona G., Caligiuri I., Canzonieri V., Toffoli G., Rizzolio F. (2016). Exosomes increase the therapeutic index of doxorubicin in breast and ovarian cancer mouse models. Nanomedicine.

[44] Saari H., Lázaro-Ibáñez E., Viitala T., Vuorimaa-Laukkanen E., Siljander P., Yliperttula M. (2015). Microvesicle- and exosome-mediated drug delivery enhances the cytotoxicity of Paclitaxel in autologous prostate cancer cells. J. Control. Release.

[45] Dreyer F., Baur A. (2016). Biogenesis and functions of exosomes and extracellular vesicles. Methods in Molecular Biology.

[46] Van Niel G., Porto-Carreiro I., Simoes S., Raposo G. (2006). Exosomes: A common pathway for a specialized function. J. Biochem..

[47] Théry C., Regnault A., Garin J., Wolfers J., Zitvogel L., Ricciardi-Castagnoli P., Raposo G., Amigorena S. (1999). Molecular characterization of dendritic cell-derived exosomes: Selective accumulation of the heat shock protein hsc73. J. Cell Biol..

[48] Davies B.A., Lee J.R.E., Oestreich A.J., Katzmann D.J. (2009). Membrane protein targeting to the MVB/lysosome. Chem. Rev..

[49] Johnstone R.M., Ahn J. (1990). A common mechanism may be involved in the selective loss of plasma membrane functions during reticulocyte maturation. Biomed. Biochim. Acta.

[50] Simons M., Raposo G. (2009). Exosomes--Vesicular carriers for intercellular communication. Curr. Opin. Cell Biol..

[51] Rusten T.E., Vaccari T., Stenmark H. (2012). Shaping development with ESCRTs. Nat. Cell Biol..

[52] Hessvik N.P., Llorente A. (2018). Current knowledge on exosome biogenesis and release. Cell. Mol. Life Sci..

[53] Schneider A., Simons M. (2013). Exosomes: Vesicular carriers for intercellular communication in neurodegenerative disorders. Cell Tissue Res..

[54] Zhang H.G. (2013). Emerging Concepts of Tumor Exosome–Mediated Cell–Cell Communication.

[55] Van Niel G., Raposo G., Candalh C., Boussac M., Hershberg R., Cerf-Bensussan N., Heyman M. (2001). Intestinal epithelial cells secrete exosome-like vesicles. Gastroenterology.

[56] Futter C.E., White I.J. (2007). Annexins and endocytosis. Traffic.

[57] Mears R., Craven R.A., Hanrahan S., Totty N., Upton C., Young S.L., Patel P., Selby P.J., Banks R.E. (2004). Proteomic analysis of melanoma-derived exosomes by two-dimensional polyacrylamide gel electrophoresis and mass spectrometry. Proteomics.

[58] Bobrie A., Colombo M., Raposo G., Théry C. (2011). Exosome Secretion: Molecular Mechanisms and Roles in Immune Responses. Traffic.

[59] Théry C., Boussac M., Véron P., Ricciardi-Castagnoli P., Raposo G., Garin J., Amigorena S. (2001). Proteomic Analysis of Dendritic Cell-Derived Exosomes: A Secreted Subcellular Compartment Distinct from Apoptotic Vesicles. J. Immunol..

[60] Mittelbrunn M., Gutiérrez-Vázquez C., Villarroya-Beltri C., González S., Sánchez-Cabo F., González M.Á., Bernad A., Sánchez-Madrid F. (2011). Unidirectional transfer of microRNA-loaded exosomes from T cells to antigen-presenting cells. Nat. Commun..

[61] Admyre C., Johansson S.M., Qazi K.R., Filen J.-J., Lahesmaa R., Norman M., Neve E.P.A., Scheynius A., Gabrielsson S., Filén J.-J. (2007). Exosomes with Immune Modulatory Features Are Present in Human Breast Milk. J. Immunol..

[62] Taylor D.D., Gercel-Taylor C. (2011). Exosomes/microvesicles: Mediators of cancer-associated immunosuppressive microenvironments. Semin. Immunopathol..

[63] Liang B., Peng P., Chen S., Li L., Zhang M., Cao D., Yang J., Li H., Gui T., Li X. (2013). Characterization and proteomic analysis of ovarian cancer-derived exosomes. J. Proteom..

[64] Henderson M.C., Azorsa D.O. (2012). The genomic and proteomic content of cancer cell-derived exosomes. Front. Oncol..

[65] Wang W., Lotze M.T. (2014). Good things come in small packages: Exosomes, immunity and cancer. Cancer Gene Ther..

[66] Robbins P.D., Morelli A.E. (2014). Regulation of immune responses by extracellular vesicles. Nat. Rev. Immunol..

[67] Devhare P.B., Ray R.B. (2017). A novel role of exosomes in the vaccination approach. Ann. Transl. Med..

[68] Lachenal G., Pernet-Gallay K., Chivet M., Hemming F.J., Belly A., Bodon G., Blot B., Haase G., Goldberg Y., Sadoul R. (2011). Release of exosomes from differentiated neurons and its regulation by synaptic glutamatergic activity. Mol. Cell. Neurosci..

[69] Ghidoni R., Benussi L., Binetti G. (2008). Exosomes: The Trojan horses of neurodegeneration. Med. Hypotheses.

[70] Jan A.T., Malik M.A., Rahman S., Yeo H.R., Lee E.J., Abdullah T.S., Choi I. (2017). Perspective insights of exosomes in neurodegenerative diseases: A critical appraisal. Front. Aging Neurosci..

[71] Zhang J., Ma J., Long K., Qiu W., Wang Y., Hu Z., Liu C., Luo Y., Jiang A., Jin L. (2017). Overexpression of exosomal cardioprotective miRNAs mitigates hypoxia-induced H9c2 cells apoptosis. Int. J. Mol. Sci..

[72] Tian C., Gao L., Zimmerman M.C., Zucker I.H. (2018). Myocardial infarction-induced microRNA-enriched exosomes contribute to cardiac Nrf2 dysregulation in chronic heart failure. Am. J. Physiol. Hear. Circ. Physiol..

[73] Taylor D.D., Gercel-Taylor C. (2008). MicroRNA signatures of tumor-derived exosomes as diagnostic biomarkers of ovarian cancer. Gynecol. Oncol..

[74] Logozzi M., De Milito A., Lugini L., Borghi M., Calabrò L., Spada M., Perdicchio M., Marino M.L., Federici C., Iessi E. (2009). High levels of exosomes expressing CD63 and caveolin-1 in plasma of melanoma patients. PLoS ONE.

[75] Record M., Subra C., Silvente-Poirot S., Poirot M. (2011). Exosomes as intercellular signalosomes and pharmacological effectors. Biochem. Pharmacol..

[76] Ostrowski M., Carmo N.B., Krumeich S., Fanget I., Raposo G., Savina A., Moita C.F., Schauer K., Hume A.N., Freitas R.P. (2010). Rab27a and Rab27b control different steps of the exosome secretion pathway. Nat. Cell Biol..

[77] Savina A., Vidal M., Colombo M.I. (2002). The exosome pathway in K562 cells is regulated by Rab11. J. Cell Sci..

[78] Hsu C., Morohashi Y., Yoshimura S.I., Manrique-Hoyos N., Jung S.Y., Lauterbach M.A., Bakhti M., Grønborg M., Möbius W., Rhee J.S. (2010). Regulation of exosome secretion by Rab35 and its GTPase-activating proteins TBC1D10A-C. J. Cell Biol..

[79] Tzeng H.T., Wang Y.C. (2016). Rab-mediated vesicle trafficking in cancer. J. Biomed. Sci..

[80] Yu X., Harris S.L., Levine A.J. (2006). The regulation of exosome secretion: A novel function of the p53 protein. Cancer Res..

[81] Record M., Carayon K., Poirot M., Silvente-Poirot S. (2014). Exosomes as new vesicular lipid transporters involved in cell-cell communication and various pathophysiologies. Biochim. Biophys. Acta.

[82] Humpton T.J., Vousden K.H. (2016). Regulation of cellular metabolism and hypoxia by p53. Cold Spring Harb. Perspect. Med..

[83] Azmi A.S., Bao B., Sarkar F.H. (2013). Exosomes in cancer development, metastasis, and drug resistance: A comprehensive review. Cancer Metastasis Rev..

[84] Yu X., Riley T., Levine A.J. (2009). The regulation of the endosomal compartment by p53 the tumor suppressor gene. FEBS J..

[85] Parolini I., Federici C., Raggi C., Lugini L., Palleschi S., De Milito A., Coscia C., Iessi E., Logozzi M., Molinari A. (2009). Microenvironmental pH is a key factor for exosome traffic in tumor cells. J. Biol. Chem..

[86] Arenaccio C., Federico M. (2017). The Multifaceted Functions of Exosomes in Health and Disease: An Overview. Adv. Exp. Med. Biol..

[87] Zhang Y., Liu Y., Liu H., Tang W.H. (2019). Exosomes: Biogenesis, biologic function and clinical potential. Cell Biosci..

[88] Mathivanan S., Fahner C.J., Reid G.E., Simpson R.J. (2012). ExoCarta 2012: Database of exosomal proteins, RNA and lipids. Nucleic Acids Res..

[89] Mulcahy L.A., Pink R.C., Carter D.R.F. (2014). Routes and mechanisms of extracellular vesicle uptake. J. Extracell. Vesicles.

[90] Mathieu M., Martin-Jaular L., Lavieu G., Théry C. (2019). Specificities of secretion and uptake of exosomes and other extracellular vesicles for cell-to-cell communication. Nat. Cell Biol..

[91] Keerthikumar S., Chisanga D., Ariyaratne D., Al Saffar H., Anand S., Zhao K., Samuel M., Pathan M., Jois M., Chilamkurti N. (2016). ExoCarta: A Web-Based Compendium of Exosomal Cargo. J. Mol. Biol..

[92] Théry C., Witwer K.W., Aikawa E., Alcaraz M.J., Anderson J.D., Andriantsitohaina R., Antoniou A., Arab T., Archer F., Atkin-Smith G.K. (2018). Minimal information for studies of extracellular vesicles 2018 (MISEV2018): A position statement of the International Society for Extracellular Vesicles and update of the MISEV2014 guidelines. J. Extracell. Vesicles.

[93] Greening D.W., Xu R., Ji H., Tauro B.J., Simpson R.J. (2015). A protocol for exosome isolation and characterization: Evaluation of ultracentrifugation, density-gradient separation, and immunoaffinity capture methods. Methods Mol. Biol..

[94] Chevillet J.R., Kang Q., Ruf I.K., Briggs H.A., Vojtech L.N., Hughes S.M., Cheng H.H., Arroyo J.D., Meredith E.K., Gallichotte E.N. (2014). Quantitative and stoichiometric analysis of the microRNA content of exosomes. Proc. Natl. Acad. Sci. USA.

[95] Lötvall J., Hill A.F., Hochberg F., Buzás E.I., Di Vizio D., Gardiner C., Gho Y.S., Kurochkin I.V., Mathivanan S., Quesenberry P. (2014). Minimal experimental requirements for definition of extracellular vesicles and their functions: A position statement from the International Society for Extracellular Vesicles. J. Extracell. Vesicles.

[96] Aalberts M., van Dissel-Emiliani F.M., van Adrichem N.P., van Wijnen M., Wauben M.H., Stout T.A., Stoorvogel W. (2012). Identification of distinct populations of prostasomes that differentially express prostate stem cell antigen, annexin A1, and GLIPR2 in humans. Biol. Reprod..

[97] Théry C., Amigorena S., Raposo G., Clayton A. (2006). Isolation and Characterization of Exosomes from Cell Culture Supernatants and Biological Fluids. Curr. Protoc. Cell Biol..

[98] Taylor D.D., Shah S. (2015). Methods of isolating extracellular vesicles impact down-stream analyses of their cargoes. Methods.

[99] Taylor D.D., Zacharias W., Gercel-Taylor C. (2011). Exosome isolation for proteomic analyses and RNA profiling. Methods Mol. Biol..

[100] Muller L., Hong C.S., Stolz D.B., Watkins S.C., Whiteside T.L. (2014). Isolation of biologically-active exosomes from human plasma. J. Immunol. Methods.

[101] Ramirez M.I., Amorim M.G., Gadelha C., Milic I., Welsh J.A., Freitas V.M., Nawaz M., Akbar N., Couch Y., Makin L. (2018). Technical challenges of working with extracellular vesicles. Nanoscale.

[102] Liga A., Vliegenthart A.D.B., Oosthuyzen W., Dear J.W., Kersaudy-Kerhoas M. (2015). Exosome isolation: A microfluidic road-map. Lab Chip.

[103] Chen C., Skog J., Hsu C.H., Lessard R.T., Balaj L., Wurdinger T., Carter B.S., Breakefield X.O., Toner M., Irimia D. (2010). Microfluidic isolation and transcriptome analysis of serum microvesicles. Lab Chip.

[104] Sparks D.L., Phillips M.C. (1992). Quantitative measurement of lipoprotein surface charge by agarose gel electrophoresis. J. Lipid Res..

[105] Wang Z., Wu H.J., Fine D., Schmulen J., Hu Y., Godin B., Zhang J.X.J., Liu X. (2013). Ciliated micropillars for the microfluidic-based isolation of nanoscale lipid vesicles. Lab Chip.

[106] Yekula A., Muralidharan K., Kang K.M., Wang L., Balaj L., Carter B.S. (2020). From laboratory to clinic: Translation of extracellular vesicle based cancer biomarkers. Methods.

[107] Taverna S., Flugy A., Saieva L., Kohn E.C., Santoro A., Meraviglia S., De Leo G., Alessandro R. (2012). Role of exosomes released by chronic myelogenous leukemia cells in angiogenesis. Int. J. Cancer.

[108] Li L., Li C., Wang S., Wang Z., Jiang J., Wang W., Li X., Chen J., Liu K., Li C. (2016). Exosomes derived from hypoxic oral squamous cell carcinoma cells deliver miR-21 to normoxic cells to elicit a prometastatic phenotype. Cancer Res..

[109] Sung B.H., Weaver A.M. (2017). Exosome secretion promotes chemotaxis of cancer cells. Cell Adhes. Migr..

[110] Logozzi M., Mizzoni D., Angelini D.F., Di Raimo R., Falchi M., Battistini L., Fais S. (2018). Microenvironmental pH and exosome levels interplay in human cancer cell lines of different histotypes. Cancers.

[111] Milane L., Singh A., Mattheolabakis G., Suresh M., Amiji M.M. (2015). Exosome mediated communication within the tumor microenvironment. J. Control. Release.

[112] Emmanouilidi A., Paladin D., Greening D.W., Falasca M. (2019). Oncogenic and Non-Malignant Pancreatic Exosome Cargo Reveal Distinct Expression of Oncogenic and Prognostic Factors Involved in Tumor Invasion and Metastasis. Proteomics.

[113] Scioli M.G., Storti G., D’amico F., Gentile P., Kim B.S., Cervelli V., Orlandi A. (2019). Adipose-derived stem cells in cancer progression: New perspectives and opportunities. Int. J. Mol. Sci..

[114] Zheng P., Luo Q., Wang W., Li J., Wang T., Wang P., Chen L., Zhang P., Chen H., Liu Y. (2018). Tumor-associated macrophages-derived exosomes promote the migration of gastric cancer cells by transfer of functional Apolipoprotein e. Cell Death Dis..

[115] Whiteside T.L. (2016). Tumor-Derived Exosomes and Their Role in Cancer Progression. Advances in Clinical Chemistry.

[116] Kalra H., Drummen G.P.C., Mathivanan S. (2016). Focus on extracellular vesicles: Introducing the next small big thing. Int. J. Mol. Sci..

[117] Kanada M., Bachmann M.H., Contag C.H. (2016). Signaling by Extracellular Vesicles Advances Cancer Hallmarks. Trends Cancer.

[118] Frydrychowicz M., Kolecka-Bednarczyk A., Madejczyk M., Yasar S., Dworacki G. (2015). Exosomes-structure, biogenesis and biological role in non-small-cell lung cancer. Scand. J. Immunol..

[119] Kamalden T.A., Macgregor-Das A.M., Kannan S.M., Dunkerly-Eyring B., Khaliddin N., Xu Z., Fusco A.P., Yazib S.A., Chow R.C., Duh E.J. (2017). Exosomal MicroRNA-15a Transfer from the Pancreas Augments Diabetic Complications by Inducing Oxidative Stress. Antioxid. Redox Signal..

[120] Mineo M., Garfield S.H., Taverna S., Flugy A., De Leo G., Alessandro R., Kohn E.C. (2012). Exosomes released by K562 chronic myeloid leukemia cells promote angiogenesis in a src-dependent fashion. Angiogenesis.

[121] Grange C., Tapparo M., Collino F., Vitillo L., Damasco C., Deregibus M.C., Tetta C., Bussolati B., Camussi G. (2011). Microvesicles released from human renal cancer stem cells stimulate angiogenesis and formation of lung premetastatic niche. Cancer Res..

[122] Sahoo S., Klychko E., Thorne T., Misener S., Schultz K.M., Millay M., Ito A., Liu T., Kamide C., Agrawal H. (2011). Exosomes from human CD34+ stem cells mediate their proangiogenic paracrine activity. Circ. Res..

[123] Deng T., Zhang H., Yang H., Wang H., Bai M., Sun W., Wang X., Si Y., Ning T., Zhang L. (2020). Exosome miR-155 Derived from Gastric Carcinoma Promotes Angiogenesis by Targeting the c-MYB/VEGF Axis of Endothelial Cells. Mol. Ther. Nucleic Acids.

[124] Tadokoro H., Umezu T., Ohyashiki K., Hirano T., Ohyashiki J.H. (2013). Exosomes derived from hypoxic leukemia cells enhance tube formation in endothelial cells. J. Biol. Chem..

[125] Webber J., Steadman R., Mason M.D., Tabi Z., Clayton A. (2010). Cancer exosomes trigger fibroblast to myofibroblast differentiation. Cancer Res..

[126] Nazarenko I., Rana S., Baumann A., McAlear J., Hellwig A., Trendelenburg M., Lochnit G., Preissner K.T., Zöller M. (2010). Cell surface tetraspanin Tspan8 contributes to molecular pathways of exosome-induced endothelial cell activation. Cancer Res..

[127] Li M., Lu Y., Xu Y., Wang J., Zhang C., Du Y., Wang L., Li L., Wang B., Shen J. (2018). Horizontal transfer of exosomal CXCR4 promotes murine hepatocarcinoma cell migration, invasion and lymphangiogenesis. Gene.

[128] Sun B., Zhou Y., Fang Y., Li Z., Gu X., Xiang J. (2019). Colorectal cancer exosomes induce lymphatic network remodeling in lymph nodes. Int. J. Cancer.

[129] Ono M., Kosaka N., Tominaga N., Yoshioka Y., Takeshita F., Takahashi R.U., Yoshida M., Tsuda H., Tamura K., Ochiya T. (2014). Exosomes from bone marrow mesenchymal stem cells contain a microRNA that promotes dormancy in metastatic breast cancer cells. Sci. Signal..

[130] Yang M., Chen J., Su F., Yu B., Su F., Lin L., Liu Y., Huang J.D., Song E. (2011). Microvesicles secreted by macrophages shuttle invasion-potentiating microRNAs into breast cancer cells. Mol. Cancer.

[131] Cooks T., Pateras I.S., Jenkins L.M., Patel K.M., Robles A.I., Morris J., Forshew T., Appella E., Gorgoulis V.G., Harris C.C. (2018). Mutant p53 cancers reprogram macrophages to tumor supporting macrophages via exosomal miR-1246. Nat. Commun..

[132] Huan J., Hornick N.I., Shurtleff M.J., Skinner A.M., Goloviznina N.A., Roberts C.T., Kurre P. (2013). RNA trafficking by acute myelogenous leukemia exosomes. Cancer Res..

[133] Peinado H., Alečković M., Lavotshkin S., Matei I., Costa-Silva B., Moreno-Bueno G., Hergueta-Redondo M., Williams C., García-Santos G., Ghajar C.M. (2012). Melanoma exosomes educate bone marrow progenitor cells toward a pro-metastatic phenotype through MET. Nat. Med..

[134] Mu W., Rana S., Zöller M. (2013). Host matrix modulation by tumor exosomes promotes motility and invasiveness. Neoplasia.

[135] Xu Q., Chiao P., Sun Y. (2016). Amphiregulin in cancer: New insights for translational medicine. Trends Cancer.

[136] Wang D., Wang X., Si M., Yang J., Sun S., Wu H., Cui S., Qu X., Yu X. (2020). Exosome-encapsulated miRNAs contribute to CXCL12/CXCR4-induced liver metastasis of colorectal cancer by enhancing M2 polarization of macrophages. Cancer Lett..

[137] Trigaux J.P., Van Beers B., Delchambre F. (1991). Male genital tract malformations associated with ipsilateral renal agenesis: Sonographic findings. J. Clin. Ultrasound.

[138] Han L., Xu J., Xu Q., Zhang B., Lam E.W.-F., Sun Y. (2017). Extracellular vesicles in the tumor microenvironment: Therapeutic resistance, clinical biomarkers, and targeting strategies. Med. Res. Rev..

[139] Ciravolo V., Huber V., Ghedini G.C., Venturelli E., Bianchi F., Campiglio M., Morelli D., Villa A., Della Mina P., Menard S. (2012). Potential role of HER2-overexpressing exosomes in countering trastuzumab-based therapy. J. Cell. Physiol..

[140] Gilbert L.A., Hemann M.T. (2011). Chemotherapeutic resistance: Surviving stressful situations. Cancer Res..

[141] Chen W.X., Cai Y.Q., Lv M.M., Chen L., Zhong S.L., Ma T.F., Zhao J.H., Tang J.H. (2014). Exosomes from docetaxel-resistant breast cancer cells alter chemosensitivity by delivering microRNAs. Tumor Biol..

[142] Chen W.X., Liu X.M., Lv M.M., Chen L., Zhao J.H., Zhong S.L., Ji M.H., Hu Q., Luo Z., Wu J.Z. (2014). Exosomes from drug-resistant breast cancer cells transmit chemoresistance by a horizontal transfer of MicroRNAs. PLoS ONE.

[143] Wang X., Zhang H., Yang H., Bai M., Ning T., Deng T., Liu R., Fan Q., Zhu K., Li J. (2020). Exosome-delivered circRNA promotes glycolysis to induce chemoresistance through the miR-122-PKM2 axis in colorectal cancer. Mol. Oncol..

[144] Corcoran C., Rani S., O’Brien K., O’Neill A., Prencipe M., Sheikh R., Webb G., McDermott R., Watson W., Crown J. (2012). Docetaxel-Resistance in Prostate Cancer: Evaluating Associated Phenotypic Changes and Potential for Resistance Transfer via Exosomes. PLoS ONE.

[145] Lv M.M., Zhu X.Y., Chen W.X., Zhong S.L., Hu Q., Ma T.F., Zhang J., Chen L., Tang J.H., Zhao J.H. (2014). Exosomes mediate drug resistance transfer in MCF-7 breast cancer cells and a probable mechanism is delivery of P-glycoprotein. Tumor Biol..

[146] Sun Y., Zhu D., Chen F., Qian M., Wei H., Chen W., Xu J. (2016). SFRP2 augments WNT16B signaling to promote therapeutic resistance in the damaged tumor microenvironment. Oncogene.

[147] Chowdhury R., Webber J.P., Gurney M., Mason M.D., Tabi Z., Clayton A. (2015). Cancer exosomes trigger mesenchymal stem cell differentiation into pro-angiogenic and pro-invasive myofibroblasts. Oncotarget.

[148] Kucharzewska P., Christianson H.C., Welch J.E., Svensson K.J., Fredlund E., Ringnér M., Mörgelin M., Bourseau-Guilmain E., Bengzon J., Belting M. (2013). Exosomes reflect the hypoxic status of glioma cells and mediate hypoxia-dependent activation of vascular cells during tumor development. Proc. Natl. Acad. Sci. USA.

[149] Horie K., Kawakami K., Fujita Y., Sugaya M., Kameyama K., Mizutani K., Deguchi T., Ito M. (2017). Exosomes expressing carbonic anhydrase 9 promote angiogenesis. Biochem. Biophys. Res. Commun..

[150] Luga V., Zhang L., Viloria-Petit A.M., Ogunjimi A.A., Inanlou M.R., Chiu E., Buchanan M., Hosein A.N., Basik M., Wrana J.L. (2012). Exosomes Mediate Stromal Mobilization of Autocrine Wnt-PCP Signaling in Breast Cancer Cell Migration. Cell.

[151] Roccaro A.M., Sacco A., Maiso P., Azab A.K., Tai Y.T., Reagan M., Azab F., Flores L.M., Campigotto F., Weller E. (2013). BM mesenchymal stromal cell-derived exosomes facilitate multiple myeloma progression. J. Clin. Investig..

[152] Zhu W., Huang L., Li Y., Zhang X., Gu J., Yan Y., Xu X., Wang M., Qian H., Xu W. (2012). Exosomes derived from human bone marrow mesenchymal stem cells promote tumor growth in vivo. Cancer Lett..

[153] Liang H., Yan X., Pan Y., Wang Y., Wang N., Li L., Liu Y., Chen X., Zhang C.Y., Gu H. (2015). MicroRNA-223 delivered by platelet-derived microvesicles promotes lung cancer cell invasion via targeting tumor suppressor EPB41L3. Mol. Cancer.

[154] Suetsugu A., Honma K., Saji S., Moriwaki H., Ochiya T., Hoffman R.M. (2013). Imaging exosome transfer from breast cancer cells to stroma at metastatic sites in orthotopic nude-mouse models. Adv. Drug Deliv. Rev..

[155] Corrado C., Raimondo S., Saieva L., Flugy A.M., De Leo G., Alessandro R. (2014). Exosome-mediated crosstalk between chronic myelogenous leukemia cells and human bone marrow stromal cells triggers an Interleukin 8-dependent survival of leukemia cells. Cancer Lett..

[156] Jenjaroenpun P., Kremenska Y., Nair V.M., Kremenskoy M., Joseph B., Kurochkin I.V. (2013). Characterization of RNA in exosomes secreted by human breast cancer cell lines using next-generation sequencing. PeerJ.

[157] Hood J.L., San R.S., Wickline S.A. (2011). Exosomes released by melanoma cells prepare sentinel lymph nodes for tumor metastasis. Cancer Res..

[158] Kruger S., Elmageed Z.Y.A., Hawke D.H., Wörner P.M., Jansen D.A., Abdel-Mageed A.B., Alt E.U., Izadpanah R. (2014). Molecular characterization of exosome-like vesicles from breast cancer cells. BMC Cancer.

[159] Atay S., Banskota S., Crow J., Sethi G., Rink L., Godwin A.K. (2014). Oncogenic KIT-containing exosomes increasegastrointestinal stromal tumor cell invasion. Proc. Natl. Acad. Sci. USA.

[160] Hoshino D., Kirkbride K.C., Costello K., Clark E.S., Sinha S., Grega-Larson N., Tyska M.J., Weaver A.M. (2013). Exosome secretion is enhanced by invadopodia and drives invasive behavior. Cell Rep..

[161] Zeng A.L., Yan W., Liu Y.W., Wang Z., Hu Q., Nie E., Zhou X., Li R., Wang X.F., Jiang T. (2017). Tumour exosomes from cells harbouring PTPRZ1-MET fusion contribute to a malignant phenotype and temozolomide chemoresistance in glioblastoma. Oncogene.

[162] Federici C., Petrucci F., Caimi S., Cesolini A., Logozzi M., Borghi M., D’Ilio S., Lugini L., Violante N., Azzarito T. (2014). Exosome release and low pH belong to a framework of resistance of human melanoma cells to cisplatin. PLoS ONE.

[163] Xiao X., Yu S., Li S., Wu J., Ma R., Cao H., Zhu Y., Feng J. (2014). Exosomes: Decreased sensitivity of lung cancer A549 cells to cisplatin. PLoS ONE.

[164] Aung T., Chapuy B., Vogel D., Wenzel D., Oppermann M., Lahmann M., Weinhage T., Menck K., Hupfeld T., Koch R. (2011). Exosomal evasion of humoral immunotherapy in aggressive B-cell lymphoma modulated by ATP-binding cassette transporter A3. Proc. Natl. Acad. Sci. USA.

[165] Chapuy B., Koch R., Radunski U., Corsham S., Cheong N., Inagaki N., Ban N., Wenzel D., Reinhardt D., Zapf A. (2008). Intracellular ABC transporter A3 confers multidrug resistance in leukemia cells by lysosomal drug sequestration. Leukemia.

[166] Zhu X., Shen H., Yin X., Yang M., Wei H., Chen Q., Feng F., Liu Y., Xu W., Li Y. (2019). Macrophages derived exosomes deliver miR-223 to epithelial ovarian cancer cells to elicit a chemoresistant phenotype. J. Exp. Clin. Cancer Res..

[167] Penfornis P., Vallabhaneni K.C., Whitt J., Pochampally R. (2016). Extracellular vesicles as carriers of microRNA, proteins and lipids in tumor microenvironment. Int. J. Cancer.

[168] Boukouris S., Mathivanan S. (2015). Exosomes in bodily fluids are a highly stable resource of disease biomarkers. Proteom. Clin. Appl..

[169] He M., Zeng Y. (2016). Microfluidic Exosome Analysis toward Liquid Biopsy for Cancer. J. Lab. Autom..

[170] Mitchell P.S., Parkin R.K., Kroh E.M., Fritz B.R., Wyman S.K., Pogosova-Agadjanyan E.L., Peterson A., Noteboom J., O’Briant K.C., Allen A. (2008). Circulating microRNAs as stable blood-based markers for cancer detection. Proc. Natl. Acad. Sci. USA.

[171] McKiernan J., Donovan M.J., O’Neill V., Bentink S., Noerholm M., Belzer S., Skog J., Kattan M.W., Partin A., Andriole G. (2016). A novel urine exosome gene expression assay to predict high-grade prostate cancer at initial biopsy. JAMA Oncol..

[172] Zhang G., Zhang W., Li B., Stringer-Reasor E., Chu C., Sun L., Bae S., Chen D., Wei S., Jiao K. (2017). MicroRNA-200c and microRNA- 141 are regulated by a FOXP3-KAT2B axis and associated with tumor metastasis in breast cancer. Breast Cancer Res..

[173] Liu J., Sun H., Wang X., Yu Q., Li S., Yu X., Gong W. (2014). Increased exosomal microRNA-21 and microRNA-146a levels in the cervicovaginal lavage specimens of patients with cervical cancer. Int. J. Mol. Sci..

[174] Ogata-Kawata H., Izumiya M., Kurioka D., Honma Y., Yamada Y., Furuta K., Gunji T., Ohta H., Okamoto H., Sonoda H. (2014). Circulating exosomal microRNAs as biomarkers of colon cancer. PLoS ONE.

[175] Manterola L., Guruceaga E., Pérez-Larraya J.G., González-Huarriz M., Jauregui P., Tejada S., Diez-Valle R., Segura V., Samprón N., Barrena C. (2014). A small noncoding RNA signature found in exosomes of GBM patient serum as a diagnostic tool. Neuro Oncol..

[176] Akers J.C., Ramakrishnan V., Kim R., Skog J., Nakano I., Pingle S., Kalinina J., Hua W., Kesari S., Mao Y. (2013). miR-21 in the Extracellular Vesicles (EVs) of Cerebrospinal Fluid (CSF): A Platform for Glioblastoma Biomarker Development. PLoS ONE.

[177] Cazzoli R., Buttitta F., Di Nicola M., Malatesta S., Marchetti A., Rom W.N., Pass H.I. (2013). MicroRNAs derived from circulating exosomes as noninvasive biomarkers for screening and diagnosing lung cancer. J. Thorac. Oncol..

[178] Rodríguez M., Silva J., López-Alfonso A., López-Muñiz M.B., Peña C., Domínguez G., García J.M., López-Gónzalez A., Méndez M., Provencio M. (2014). Different exosome cargo from plasma/bronchoalveolar lavage in non-small-cell lung cancer. Genes Chromosom. Cancer.

[179] Que R., Ding G., Chen J., Cao L. (2013). Analysis of serum exosomal microRNAs and clinicopathologic features of patients with pancreatic adenocarcinoma. World J. Surg. Oncol..

[180] Madhavan B., Yue S., Galli U., Rana S., Gross W., Müller M., Giese N.A., Kalthoff H., Becker T., Büchler M.W. (2015). Combined evaluation of a panel of protein and miRNA serum-exosome biomarkers for pancreatic cancer diagnosis increases sensitivity and specificity. Int. J. Cancer.

[181] Bryant R.J., Pawlowski T., Catto J.W.F., Marsden G., Vessella R.L., Rhees B., Kuslich C., Visakorpi T., Hamdy F.C. (2012). Changes in circulating microRNA levels associated with prostate cancer. Br. J. Cancer.

[182] Nilsson J., Skog J., Nordstrand A., Baranov V., Mincheva-Nilsson L., Breakefield X.O., Widmark A. (2009). Prostate cancer-derived urine exosomes: A novel approach to biomarkers for prostate cancer. Br. J. Cancer.

[183] Skog J., Würdinger T., van Rijn S., Meijer D.H., Gainche L., Curry W.T., Carter B.S., Krichevsky A.M., Breakefield X.O. (2008). Glioblastoma microvesicles transport RNA and proteins that promote tumour growth and provide diagnostic biomarkers. Nat. Cell Biol..

[184] Shao H., Chung J., Balaj L., Charest A., Bigner D.D., Carter B.S., Hochberg F.H., Breakefield X.O., Weissleder R., Lee H. (2012). Protein typing of circulating microvesicles allows real-time monitoring of glioblastoma therapy. Nat. Med..

[185] Klibi J., Niki T., Riedel A., Pioche-Durieu C., Souquere S., Rubinstein E., Moulec S.L.E., Guigay J., Hirashima M., Guemira F. (2009). Blood diffusion and Th1-suppressive effects of galectin-9-containing exosomes released by Epstein-Barr virus-infected nasopharyngeal carcinoma cells. Blood.

[186] Houali K., Wang X., Shimizu Y., Djennaoui D., Nicholls J., Fiorini S., Bouguermouh A., Ooka T. (2007). A new diagnostic marker for secreted Epstein-Barr virus-encoded LMP1 and BARF1 oncoproteins in the serum and saliva of patients with nasopharyngeal carcinoma. Clin. Cancer Res..

[187] Guan M., Chen X., Ma Y., Tang L., Guan L., Ren X., Yu B., Zhang W., Su B. (2015). MDA-9 and GRP78 as potential diagnostic biomarkers for early detection of melanoma metastasis. Tumor Biol..

[188] Toth B., Nieuwland R., Liebhardt S., Ditsch N., Steinig K., Stieber P., Rank A., Göhring P., Thaler C.J., Friese K. (2008). Circulating microparticles in breast cancer patients: A comparative analysis with established biomarkers. Anticancer Res..

[189] Khan S., Bennit H.F., Turay D., Perez M., Mirshahidi S., Yuan Y., Wall N.R. (2014). Early diagnostic value of survivin and its alternative splice variants in breast cancer. BMC Cancer.

[190] Li Y., Zhang Y., Qiu F., Qiu Z. (2011). Proteomic identification of exosomal LRG1: A potential urinary biomarker for detecting NSCLC. Electrophoresis.

[191] Yamashita T., Kamada H., Kanasaki S., Maeda Y., Nagano K., Abe Y., Inoue M., Yoshioka Y., Tsutsumi Y., Katayama S. (2013). Epidermal growth factor receptor localized to exosome membranes as a possible biomarker for lung cancer diagnosis. Pharmazie.

[192] Sandfeld-Paulsen B., Aggerholm-Pedersen N., Bæk R., Jakobsen K.R., Meldgaard P., Folkersen B.H., Rasmussen T.R., Varming K., Jørgensen M.M., Sorensen B.S. (2016). Exosomal proteins as prognostic biomarkers in non-small cell lung cancer. Mol. Oncol..

[193] Ueda K., Ishikawa N., Tatsuguchi A., Saichi N., Fujii R., Nakagawa H. (2014). Antibody-coupled monolithic silica microtips for highthroughput molecular profiling of circulating exosomes. Sci. Rep..

[194] Costa-Silva B., Aiello N.M., Ocean A.J., Singh S., Zhang H., Thakur B.K., Becker A., Hoshino A., Mark M.T., Molina H. (2015). Pancreatic cancer exosomes initiate pre-metastatic niche formation in the liver. Nat. Cell Biol..

[195] Baran J., Baj-Krzyworzeka M., Weglarczyk K., Szatanek R., Zembela M., Barbasz J., Czupryna A., Szczepanik A., Zembala M. (2010). Circulating tumour-derived microvesicles in plasma of gastric cancer patients. Cancer Immunol. Immunother..

[196] Yoshioka Y., Kosaka N., Konishi Y., Ohta H., Okamoto H., Sonoda H., Nonaka R., Yamamoto H., Ishii H., Mori M. (2014). Ultra-sensitive liquid biopsy of circulating extracellular vesicles using ExoScreen. Nat. Commun..

[197] Li J., Sherman-Baust C.A., Tsai-Turton M., Bristow R.E., Roden R.B., Morin P.J. (2009). Claudin-containing exosomes in the peripheral circulation of women with ovarian cancer. BMC Cancer.

[198] Magdalena Derbis M.S. (2012). Exosomes in Plasma of Patients with Ovarian Carcinoma: Potential Biomarkers of Tumor Progression and Response to Therapy. Gynecol. Obstet..

[199] Zhao Z., Yang Y., Zeng Y., He M. (2016). A microfluidic ExoSearch chip for multiplexed exosome detection towards blood-based ovarian cancer diagnosis. Lab Chip.

[200] Keller S., König A.K., Marmé F., Runz S., Wolterink S., Koensgen D., Mustea A., Sehouli J., Altevogt P. (2009). Systemic presence and tumor-growth promoting effect of ovarian carcinoma released exosomes. Cancer Lett..

[201] Khan S., Jutzy J.M.S., Valenzuela M.M.A., Turay D., Aspe J.R., Ashok A., Mirshahidi S., Mercola D., Lilly M.B., Wall N.R. (2012). Plasma-Derived Exosomal Survivin, a Plausible Biomarker for Early Detection of Prostate Cancer. PLoS ONE.

[202] Chen C.L., Lai Y.F., Tang P., Chien K.Y., Yu J.S., Tsai C.H., Chen H.W., Wu C.C., Chung T., Hsu C.W. (2012). Comparative and targeted proteomic analyses of urinary microparticles from bladder cancer and hernia patients. J. Proteome Res..

[203] Raimondo F., Morosi L., Corbetta S., Chinello C., Brambilla P., Della Mina P., Villa A., Albo G., Battaglia C., Bosari S. (2013). Differential protein profiling of renal cell carcinoma urinary exosomes. Mol. Biosyst..

[204] Witwer K.W., Buzás E.I., Bemis L.T., Bora A., Lässer C., Lötvall J., Nolte-’t Hoen E.N., Piper M.G., Sivaraman S., Skog J. (2013). Standardization of sample collection, isolation and analysis methods in extracellular vesicle research. J. Extracell. Vesicles.

[205] Issadore D., Min C., Liong M., Chung J., Weissleder R., Lee H. (2011). Miniature magnetic resonance system for point-of-care diagnostics. Lab Chip.

[206] Orozco A.F., Lewis D.E. (2010). Flow cytometric analysis of circulating microparticles in plasma. Cytom. Part A.

[207] Im H., Shao H., Park Y.I., Peterson V.M., Castro C.M., Weissleder R., Lee H. (2014). Label-free detection and molecular profiling of exosomes with a nano-plasmonic sensor. Nat. Biotechnol..

[208] Jørgensen M., Bæk R., Pedersen S., Søndergaard E.K.L., Kristensen S.R., Varming K. (2013). Extracellular Vesicle (EV) array: Microarray capturing of exosomes and other extracellular vesicles for multiplexed phenotyping. J. Extracell. Vesicles.

[209] An T., Qin S., Xu Y., Tang Y., Huang Y., Situ B., Inal J.M., Zheng L. (2015). Exosomes serve as tumour markers for personalized diagnostics owing to their important role in cancer metastasis. J. Extracell. Vesicles.

[210] Kanwar S.S., Dunlay C.J., Simeone D.M., Nagrath S. (2014). Microfluidic device (ExoChip) for on-chip isolation, quantification and characterization of circulating exosomes. Lab Chip.

[211] Jayaseelan V.P. (2020). Emerging role of exosomes as promising diagnostic tool for cancer. Cancer Gene Ther..

[212] Liu Y., Li D., Liu Z., Zhou Y., Chu D., Li X., Jiang X., Hou D., Chen X., Chen Y. (2015). Targeted exosome-mediated delivery of opioid receptor Mu siRNA for the treatment of morphine relapse. Sci. Rep..

[213] Pascucci L., Coccè V., Bonomi A., Ami D., Ceccarelli P., Ciusani E., Viganò L., Locatelli A., Sisto F., Doglia S.M. (2014). Paclitaxel is incorporated by mesenchymal stromal cells and released in exosomes that inhibit in vitro tumor growth: A new approach for drug delivery. J. Control. Release.

[214] Yang Y., Chen Y., Zhang F., Zhao Q., Zhong H. (2015). Increased anti-tumour activity by exosomes derived from doxorubicin-treated tumour cells via heat stress. Int. J. Hyperth..

[215] Sun D., Zhuang X., Xiang X., Liu Y., Zhang S., Liu C., Barnes S., Grizzle W., Miller D., Zhang H.G. (2010). A novel nanoparticle drug delivery system: The anti-inflammatory activity of curcumin is enhanced when encapsulated in exosomes. Mol. Ther..

[216] Zhuang X., Xiang X., Grizzle W., Sun D., Zhang S., Axtell R.C., Ju S., Mu J., Zhang L., Steinman L. (2011). Treatment of brain inflammatory diseases by delivering exosome encapsulated anti-inflammatory drugs from the nasal region to the brain. Mol. Ther..

[217] Tian T., Zhu Y.L., Zhou Y.Y., Liang G.F., Wang Y.Y., Hu F.H., Xiao Z.D. (2014). Exosome uptake through clathrin-mediated endocytosis and macropinocytosis and mediating miR-21 delivery. J. Biol. Chem..

[218] Yousefpour P., Chilkoti A. (2014). Co-opting biology to deliver drugs. Biotechnol. Bioeng..

[219] Sancho-Albero M., Navascués N., Mendoza G., Sebastián V., Arruebo M., Martín-Duque P., Santamaría J. (2019). Exosome origin determines cell targeting and the transfer of therapeutic nanoparticles towards target cells. J. Nanobiotechnol..

[220] Vader P., Mol E.A., Pasterkamp G., Schiffelers R.M. (2016). Extracellular vesicles for drug delivery. Adv. Drug Deliv. Rev..

[221] Redman C.W.G., Tannetta D.S., Dragovic R.A., Gardiner C., Southcombe J.H., Collett G.P., Sargent I.L. (2012). Review: Does size matter? Placental debris and the pathophysiology of pre-eclampsia. Placenta.

[222] Rupert D.L.M., Claudio V., Lässer C., Bally M. (2017). Methods for the physical characterization and quantification of extracellular vesicles in biological samples. Biochim. Biophys. Acta Gen. Subj..

[223] Liu C., Su C. (2019). Design strategies and application progress of therapeutic exosomes. Theranostics.

[224] Wahlgren J., Karlson T.D.L., Brisslert M., Vaziri Sani F., Telemo E., Sunnerhagen P., Valadi H. (2012). Plasma exosomes can deliver exogenous short interfering RNA to monocytes and lymphocytes. Nucleic Acids Res..

[225] Kalra H., Adda C.G., Liem M., Ang C.S., Mechler A., Simpson R.J., Hulett M.D., Mathivanan S. (2013). Comparative proteomics evaluation of plasma exosome isolation techniques and assessment of the stability of exosomes in normal human blood plasma. Proteomics.

[226] Ha D., Yang N., Nadithe V. (2016). Exosomes as therapeutic drug carriers and delivery vehicles across biological membranes: Current perspectives and future challenges. Acta Pharm. Sin. B.

[227] Antimisiaris S.G., Mourtas S., Marazioti A. (2018). Exosomes and Exosome-Inspired Vesicles for Targeted Drug Delivery. Pharmaceutics.

[228] Kalani A., Kamat P.K., Chaturvedi P., Tyagi S.C., Tyagi N. (2014). Curcumin-primed exosomes mitigate endothelial cell dysfunction during hyperhomocysteinemia. Life Sci..

[229] Aqil F., Munagala R., Jeyabalan J., Agrawal A.K., Gupta R. (2017). Exosomes for the Enhanced Tissue Bioavailability and Efficacy of Curcumin. AAPS J..

[230] Yang T., Martin P., Fogarty B., Brown A., Schurman K., Phipps R., Yin V.P., Lockman P., Bai S. (2015). Exosome delivered anticancer drugs across the blood-brain barrier for brain cancer therapy in Danio Rerio. Pharm. Res..

[231] Zhao Y., Haney M.J., Gupta R., Bohnsack J.P., He Z., Kabanov A.V., Batrakova E.V. (2014). GDNF-transfected macrophages produce potent neuroprotective effects in parkinson’s disease mouse model. PLoS ONE.

[232] Zeelenberg I.S., Ostrowski M., Krumeich S., Bobrie A., Jancic C., Boissonnas A., Delcayre A., Le Pecq J.B., Combadière B., Amigorena S. (2008). Targeting tumor antigens to secreted membrane vesicles in vivo induces efficient antitumor immune responses. Cancer Res..

[233] Haney M.J., Zhao Y., Harrison E.B., Mahajan V., Ahmed S., He Z., Suresh P., Hingtgen S.D., Klyachko N.L., Mosley R.L. (2013). Specific Transfection of Inflamed Brain by Macrophages: A New Therapeutic Strategy for Neurodegenerative Diseases. PLoS ONE.

[234] Maguire C.A., Balaj L., Sivaraman S., Crommentuijn M.H.W., Ericsson M., Mincheva-Nilsson L., Baranov V., Gianni D., Tannous B.A., Sena-Esteves M. (2012). Microvesicle-associated AAV vector as a novel gene delivery system. Mol. Ther..

[235] Lv L.H., Wan Y.L., Lin Y., Zhang W., Yang M., Li G.N., Lin H.M., Shang C.Z., Chen Y.J., Min J. (2012). Anticancer drugs cause release of exosomes with heat shock proteins from human hepatocellular carcinoma cells that elicit effective natural killer cell antitumor responses in vitro. J. Biol. Chem..

[236] Kim M.S., Haney M.J., Zhao Y., Yuan D., Deygen I., Klyachko N.L., Kabanov A.V., Batrakova E.V. (2018). Engineering macrophage-derived exosomes for targeted paclitaxel delivery to pulmonary metastases: In vitro and in vivo evaluations. Nanomed. Nanotechnol. Biol. Med..

[237] Lee J., Kim J., Jeong M., Lee H., Goh U., Kim H., Kim B., Park J.H. (2015). Liposome-based engineering of cells to package hydrophobic compounds in membrane vesicles for tumor penetration. Nano Lett..

[238] EL Andaloussi S., Lakhal S., Mäger I., Wood M.J.A. (2013). Exosomes for targeted siRNA delivery across biological barriers. Adv. Drug Deliv. Rev..

[239] Shtam T.A., Kovalev R.A., Varfolomeeva E.Y., Makarov E.M., Kil Y.V., Filatov M.V. (2013). Exosomes are natural carriers of exogenous siRNA to human cells in vitro. Cell Commun. Signal..

[240] Théry C., Ostrowski M., Segura E. (2009). Membrane vesicles as conveyors of immune responses. Nat. Rev. Immunol..

[241] Lamichhane T.N., Jeyaram A., Patel D.B., Parajuli B., Livingston N.K., Arumugasaamy N., Schardt J.S., Jay S.M. (2016). Oncogene Knockdown via Active Loading of Small RNAs into Extracellular Vesicles by Sonication. Cell. Mol. Bioeng..

[242] Zhang D., Lee H., Zhu Z., Minhas J.K., Jin Y. (2016). Enrichment of selective miRNAs in exosomes and delivery of exosomal miRNAs in vitro and in vivo. Am. J. Physiol.—Lung Cell. Mol. Physiol..

[243] Kooijmans S.A.A., Stremersch S., Braeckmans K., De Smedt S.C., Hendrix A., Wood M.J.A., Schiffelers R.M., Raemdonck K., Vader P. (2013). Electroporation-induced siRNA precipitation obscures the efficiency of siRNA loading into extracellular vesicles. J. Control. Release.

[244] Lamichhane T.N., Raiker R.S., Jay S.M. (2015). Exogenous DNA loading into extracellular vesicles via electroporation is size-dependent and enables limited gene delivery. Mol. Pharm..

[245] Suwaki N., Klare K., Tarsounas M. (2011). RAD51 paralogs: Roles in DNA damage signalling, recombinational repair and tumorigenesis. Semin. Cell Dev. Biol..

[246] Liu Y., Zhao L., Li D., Yin Y., Zhang C.Y., Li J., Zhang Y. (2013). Microvesicle-delivery miR-150 promotes tumorigenesis by up-regulating VEGF, and the neutralization of miR-150 attenuate tumor development. Protein Cell.

[247] Kamerkar S., Lebleu V.S., Sugimoto H., Yang S., Ruivo C.F., Melo S.A., Lee J.J., Kalluri R. (2017). Exosomes facilitate therapeutic targeting of oncogenic KRAS in pancreatic cancer. Nature.

[248] Yim N., Ryu S.W., Choi K., Lee K.R., Lee S., Choi H., Kim J., Shaker M.R., Sun W., Park J.H. (2016). Exosome engineering for efficient intracellular delivery of soluble proteins using optically reversible protein-protein interaction module. Nat. Commun..

[249] Haney M.J., Suresh P., Zhao Y., Kanmogne G.D., Kadiu I., Sokolsky-Papkov M., Klyachko N.L., Mosley R.L., Kabanov A.V., Gendelman H.E. (2012). Blood-borne macrophage-neural cell interactions hitchhike on endosome networks for cell-based nanozyme brain delivery. Nanomedicine.

[250] Aspe J.R., Osterman C.J.D., Jutzy J.M.S., Deshields S., Whang S., Wall N.R. (2014). Enhancement of Gemcitabine sensitivity in pancreatic adenocarcinoma by novel exosome-mediated delivery of the Survivin-T34A mutant. J. Extracell. Vesicles.

[251] Nie W., Wu G., Zhang J., Huang L.L., Ding J., Jiang A., Zhang Y., Liu Y., Li J., Pu K. (2020). Responsive Exosome Nano-bioconjugates for Synergistic Cancer Therapy. Angew. Chem. Int. Ed..

[252] Escudier B., Dorval T., Chaput N., André F., Caby M.P., Novault S., Flament C., Leboulaire C., Borg C., Amigorena S. (2005). Vaccination of metastatic melanoma patients with autologous dendritic cell (DC) derived-exosomes: Results of the first phase 1 clinical trial. J. Transl. Med..

[253] André F., Chaput N., Schartz N.E.C., Flament C., Aubert N., Bernard J., Lemonnier F., Raposo G., Escudier B., Hsu D.-H. (2004). Exosomes as Potent Cell-Free Peptide-Based Vaccine. I. Dendritic Cell-Derived Exosomes Transfer Functional MHC Class I/Peptide Complexes to Dendritic Cells. J. Immunol..

[254] Morse M.A., Garst J., Osada T., Khan S., Hobeika A., Clay T.M., Valente N., Shreeniwas R., Sutton M.A., Delcayre A. (2005). A phase I study of dexosome immunotherapy in patients with advanced non-small cell lung cancer. J. Transl. Med..

[255] Dai S., Wei D., Wu Z., Zhou X., Wei X., Huang H., Li G. (2008). Phase I clinical trial of autologous ascites-derived exosomes combined with GM-CSF for colorectal cancer. Mol. Ther..

[256] Wang Q., Zhuang X., Mu J., Deng Z.B., Jiang H., Xiang X., Wang B., Yan J., Miller D., Zhang H.G. (2013). Delivery of therapeutic agents by nanoparticles made of grapefruit-derived lipids. Nat. Commun..

[257] Mu J., Zhuang X., Wang Q., Jiang H., Deng Z.B., Wang B., Zhang L., Kakar S., Jun Y., Miller D. (2014). Interspecies communication between plant and mouse gut host cells through edible plant derived exosome-like nanoparticles. Mol. Nutr. Food Res..

[258] Kumar V., Palazzolo S., Bayda S., Corona G., Toffoli G., Rizzolio F. (2016). DNA nanotechnology for cancer therapy. Theranostics.

[259] Bayda S., Hadla M., Palazzolo S., Riello P., Corona G., Toffoli G., Rizzolio F. (2017). Inorganic Nanoparticles for Cancer Therapy: A Transition from Lab to Clinic. Curr. Med. Chem..

[260] Palazzolo S., Bayda S., Hadla M., Caligiuri I., Corona G., Toffoli G., Rizzolio F. (2017). The Clinical Translation of Organic Nanomaterials for Cancer Therapy: A Focus on Polymeric Nanoparticles, Micelles, Liposomes and Exosomes. Curr. Med. Chem..

[261] Subbiah V., Grilley-Olson J.E., Combest A.J., Sharma N., Tran R.H., Bobe I., Osada A., Takahashi K., Balkissoon J., Camp A. (2018). Phase Ib/II trial of NC-6004 (nanoparticle cisplatin) plus gemcitabine in patients with advanced solid tumors. Clin. Cancer Res..

[262] Von Hoff D.D., Mita M.M., Ramanathan R.K., Weiss G.J., Mita A.C., Lorusso P.M., Burris H.A., Hart L.L., Low S.C., Parsons D.M. (2016). Phase I study of PSMA-targeted docetaxel-containing nanoparticle BIND-014 in patients with advanced solid tumors. Clin. Cancer Res..

[263] Wu S.Y., Lopez-Berestein G., Calin G.A., Sood A.K. (2014). RNAi therapies: Drugging the undruggable. Sci. Transl. Med..

[264] Fuhrmann G., Chandrawati R., Parmar P.A., Keane T.J., Maynard S.A., Bertazzo S., Stevens M.M. (2018). Engineering Extracellular Vesicles with the Tools of Enzyme Prodrug Therapy. Adv. Mater..

[265] Bayda S., Hadla M., Palazzolo S., Kumar V., Caligiuri I., Ambrosi E., Pontoglio E., Agostini M., Tuccinardi T., Benedetti A. (2017). Bottom-up synthesis of carbon nanoparticles with higher doxorubicin efficacy. J. Control. Release.

[266] Rosenblum D., Joshi N., Tao W., Karp J.M., Peer D. (2018). Progress and challenges towards targeted delivery of cancer therapeutics. Nat. Commun..

[267] Ludwig A.K., Giebel B. (2012). Exosomes: Small vesicles participating in intercellular communication. Int. J. Biochem. Cell Biol..

[268] Sharma A., Khatun Z., Shiras A. (2016). Tumor exosomes: Cellular postmen of cancer diagnosis and personalized therapy. Nanomedicine.

[269] Diaz-Cano S.J. (2012). Tumor heterogeneity: Mechanisms and bases for a reliable application of molecular marker design. Int. J. Mol. Sci..

[270] Jalalian S.H., Ramezani M., Jalalian S.A., Abnous K., Taghdisi S.M. (2019). Exosomes, new biomarkers in early cancer detection. Anal. Biochem..

[271] Wulfkuhle J.D., Liotta L.A., Petricoin E.F. (2003). Proteomic applications for the early detection of cancer. Nat. Rev. Cancer.

